# Decellularized silk fibroin scaffold primed with adipose mesenchymal stromal cells improves wound healing in diabetic mice

**DOI:** 10.1186/scrt396

**Published:** 2014-01-14

**Authors:** Stefania Elena Navone, Luisa Pascucci, Marta Dossena, Anna Ferri, Gloria Invernici, Francesco Acerbi, Silvia Cristini, Gloria Bedini, Valentina Tosetti, Valentina Ceserani, Arianna Bonomi, Augusto Pessina, Giuliano Freddi, Antonio Alessandrino, Piero Ceccarelli, Rolando Campanella, Giovanni Marfia, Giulio Alessandri, Eugenio Agostino Parati

**Affiliations:** 1 The Cellular Neurobiology Laboratory, Cerebrovascular Diseases Unit, IRCCS Foundation Neurological Institute “C. Besta”, Milan, Italy; 2 Department of Veterinary Medicine, University of Perugia, Perugia, Italy; 3 Neurosurgery Department, IRCCS Foundation Neurological Institute “C. Besta”, Milan, Italy; 4 Department of Public Health, Microbiology, Virology, University of Milan, Milan, Italy; 5 Innovhub-SSI, Div. Stazione Sperimentale per la Seta, Milan, Italy; 6 Neurosurgery Department, S. Carlo Borromeo Hospital, Milan, Italy; 7 Laboratory of Experimental Neurosurgery and Cell Therapy, Neurosurgery Unit, Fondazione IRCCS Ca’ Granda Ospedale Maggiore Policlinico Milano, Milan, Italy; 8 Current address: Laboratory of Experimental Neurosurgery and Cell Therapy, Neurosurgery Unit, Fondazione IRCCS Ca’ Granda Ospedale Maggiore Policlinico Milano, via Francesco Sforza, 28 20122 Milan, Italy

## Abstract

**Introduction:**

Silk fibroin (SF) scaffolds have been shown to be a suitable substrate for tissue engineering and to improve tissue regeneration when cellularized with mesenchymal stromal cells (MSCs). We here demonstrate, for the first time, that electrospun nanofibrous SF patches cellularized with human adipose-derived MSCs (Ad-MSCs-SF), or decellularized (D-Ad-MSCs-SF), are effective in the treatment of skin wounds, improving skin regeneration in *db/db* diabetic mice.

**Methods:**

The conformational and structural analyses of SF and D-Ad-MSCs-SF patches were performed by scanning electron microscopy, confocal microscopy, Fourier transform infrared spectroscopy and differential scanning calorimetry. Wounds were performed by a 5 mm punch biopsy tool on the mouse’s back. Ad-MSCs-SF and D-Ad-MSCs-SF patches were transplanted and the efficacy of treatments was assessed by measuring the wound closure area, by histological examination and by gene expression profile. We further investigated the *in vitro* angiogenic properties of Ad-MSCs-SF and D-Ad-MSCs-SF patches by affecting migration of human umbilical vein endothelial cells (HUVECs), keratinocytes (KCs) and dermal fibroblasts (DFs), through the aortic ring assay and, finally, by evaluating the release of angiogenic factors.

**Results:**

We found that Ad-MSCs adhere and grow on SF, maintaining their phenotypic mesenchymal profile and differentiation capacity. Conformational and structural analyses on SF and D-Ad-MSCs-SF samples, showed that sterilization, decellularization, freezing and storing did not affect the SF structure. When grafted in wounds of diabetic mice, both Ad-MSCs-SF and D-Ad-MSCs-SF significantly improved tissue regeneration, reducing the wound area respectively by 40% and 35%, within three days, completing the process in around 10 days compared to 15–17 days of controls. RT^2^ gene profile analysis of the wounds treated with Ad-MSCs-SF and D-Ad-MSCs-SF showed an increment of genes involved in angiogenesis and matrix remodeling. Finally, Ad-MSCs-SF and D-Ad-MSCs-SF co-cultured with HUVECs, DFs and KCs, preferentially enhanced the HUVECs’ migration and the release of angiogenic factors stimulating microvessel outgrowth in the aortic ring assay.

**Conclusions:**

Our results highlight for the first time that D-Ad-MSCs-SF patches are almost as effective as Ad-MSCs-SF patches in the treatment of diabetic wounds, acting through a complex mechanism that involves stimulation of angiogenesis. Our data suggest a potential use of D-Ad-MSCs-SF patches in chronic diabetic ulcers in humans.

## Introduction

Impaired wound healing is a major clinical problem in diabetic patients. A definitive pharmacological treatment of this pathological condition is currently unavailable and this often leads to limb amputation [1].

Cell-based therapies are slowly gaining ground in routine medical care and, especially, in wound management of skin. They offer promise for the repair and/or replacement of damaged tissue and the restoration of lost functionality because they possess many of the criteria necessary for wound healing [2,3].

Reports increasingly suggest that multipotent mesenchymal stromal cells (MSCs), isolated from different tissue sources, confer benefits *in vivo* as tissue restorative agents. They act by either releasing trophic factors or through their multilineage differentiation properties [3,4]. In particular, adipose tissue derived MSCs (Ad-MSCs) are currently under investigation to treat skin wounds [5] because of their abundance, easy isolation and *in vitro* expansion.

Recently, several different biosynthetic scaffolds, both bioresorbable and non-resorbable, have been used alone or in combination with cells to treat wounds [6-9]. Among the different scaffolds, silk fibroin (SF), loaded with MSCs, has been shown to possess some efficacy in repairing experimental wounds of the skin [10,11]. However, the application of scaffolds loaded with live cells *in vivo* may have some risk, due to the possible transfer of pathogens or the induction of adverse immunological reaction in the host, particularly when allogenic or xenogenic cells are utilized for therapy [12-14].

For these reasons, decellularized scaffolds have also received significant interest for tissue engineering applications [14-19]. It has been suggested that decellularized biological scaffolds may mimic a natural extracellular matrix (ECM) substrate. Moreover, decellularized scaffolds previously coated with cells or cultured cell-derived ECM scaffolds, once transplanted *in vivo*, may mimic the presence of living cells, possibly through a slow release process of cell products which have remained entrapped in the scaffold’s mesh [15-20]. Furthermore, the methods to remove the cells from the scaffold, in general, alter neither the three-dimensional native architecture of the scaffold nor the cell products [20,21].

In this study, we wanted to investigate the efficacy of SF patches, cellularized with human Ad-MSCs (Ad-MSCs-SF) or decellularized (D-Ad-MSCs-SF) by using distilled water, on experimental skin wounds in diabetic mice (*db/db* Leprdb/J). These mice carry a mutation in the leptin receptor that determines a significant delay in wound healing similar to that observed in diabetic patients [22].

We assessed the efficacy of treatments by measuring the wound closure area and by histological examination. Concomitantly, we investigated the gene expression in the regenerating tissue by RT^2^ profile, as well as the *in vitro* capacity of cellularized and decellularized SF patches to affect migration of human umbilical vein endothelial cells (HUVECs), keratinocytes (KCs) and dermal fibroblasts (DFs) and their angiogenic properties.

Our data show, for the first time, that D-Ad-MSCs-SF patches are almost as effective in the treatment of skin wounds as the cellularized ones.

## Methods

### Cell isolation and culture

The study was approved by the local institutional review board of the IRCCS Foundation Neurological Institute ‘C. Besta’, Milan Italy and informed written consent was obtained from all volunteers.

Specimens of fat from the periumbilical regions of four volunteers were collected by a lipoaspirate technique. Ad-MSCs were obtained as previously described [23]. The cells were cultured in stem cell medium (SCM), consisting of (D)MEM/F12 supplemented with 10% fetal bovine serum (Gibco, Grand Island, NY, USA), 1% penicillin and streptomycin solution (Sigma-Aldrich, Basel, Switzerland) and optimized for the growth of stem cells as previously described [24]. The Ad-MSCs were routinely seeded at 2 × 10^4^ cells/cm^2^ in T75-cm^2^ flasks and passed weekly. For experiments, the cells were not used after six passages. Cell viability was assessed by the trypan blue dye-exclusion assay.

### Flow cytometric analysis

Flow cytometry (FC) was performed on Ad-MSCs to evaluate mesenchymal, hematopoietic, endothelial and immunological markers: CD14, CD31, CD34, CD45, CD73, HLA-DR (BD Pharmingen, San Jose, CA, USA), CD105 (AbDSerotec, Raleigh, NC, USA), CD90 (Millipore, Temecula, CA, USA) and CD19 (Beckman Coulter, Milan, Italy). Briefly, 5 × 10^4^ cells/tube were stained with fluorochrome conjugated monoclonal antibodies (mAbs) and incubated for 30 minutes at 4°C in the dark. Samples were centrifuged at 300 × g for five minutes, washed twice with PBS and fixed with 4% paraformaldehyde (PFA) (Sigma-Aldrich). Analyses were performed in a scan flow cytometer by means of Cell Quest software (BD Pharmingen). Isotype-matched mouse immunoglobulins were used as control. At least 2 × 10^4^ events were acquired for each sample. Non-viable cells were excluded by physical gating.

### Multipotent differentiation potential of Ad-MSCs before and after seeding on SF patches

According to the minimal criteria suggested by Dominici *et al.*[25], Ad-MSCs were tested for their capacity to differentiate into adipocytes, chondrocytes and osteocytes, before and after seeding on SF scaffold. Briefly, to test adipogenic differentiation ability, 3.7 × 10^4^ cells/well were plated onto micro cover glasses (Zeus Scientific, Branchburg, NJ, USA) or onto SF patch in 24 multi-well plates (Sigma-Aldrich) with 0.5 mL/well of SCM. After 48 hours of incubation at 37°C, 5% CO_2,_ the culture medium was replaced with adipogenesis differentiation medium (ADM). After 21 days of culture, cells were washed two times with D-PBS, fixed for 30 minutes at room temperature with 4% PFA and stained with Oil Red O (Sigma-Aldrich) [26].

Osteogenic differentiation was obtained by incubating cells in osteogenesis differentiation medium (ODM) for 21 days. Cultured monolayers were fixed for five minutes in cooled ethanol at 70% and then processed for Alizarin Red staining (Sigma-Aldrich) [26].

Chondrogenic differentiation was performed using a Human Mesenchymal Stem Cell Functional Identification kit (R&D System, Minneapolis, USA) following the manufacturer’s instructions. After 21 days of incubation in chondrogenesis differentiation medium (CDM), the pellet was fixed for 30 minutes at room temperature with 4% PFA, cryoprotected with 30% sucrose (Sigma Aldrich) in D-PBS, embedded in optimal cutting temperature (OCT) compound (Sakura Finitek, Torrance CA, USA) and sectioned using a cryostat (Leica). The slides were processed for immunocytochemical detection of aggrecan. Negative controls (cells in SCM culture medium) were performed to evaluate the specificity of the antibody.

Histological and immunohistochemistry stainings were performed on Ad-MSCs-SF after 21 days of adipogenic, osteogenic and chondrogenic differentiation culture conditions in ADM, ODM and CDM, respectively. Briefly, the Ad-MSCs-SF patches were fixed for 30 minutes at 4°C in 4% PFA in 0.1 M D-PBS and cryoprotected with 30% sucrose (Sigma Aldrich) in D-PBS, embedded in OCT compound (Sakura Finitek) and sectioned on a cryostat (Leica). Oil Red O, Alizarin Red (Sigma-Aldrich) and aggrecan (1:100, R&D System) stainings were used to confirm adipogenic, osteogenic and chondrogenic differentiation capabilities.

### Preparation of Ad-MSCs- SF and D-Ad-MSCs-SF patches

Sheets of electrospun SF were produced according to the method of Alessandrino *et al*. [27]. Five-mm-diameter SF patches were cut by using a sterile 5-mm punch biopsy tool (KLS Martin cod. 28-240-05-07 Umkirch, Germany), placed into Petri dishes, washed with 70% ethanol solution for one hour, sterilized under UV light for six hours and then stored at 4°C until use.

Each patch was placed into a well of a 96-multiwell plate (Corning Incorporated, Corning, NY, USA) and seeded with a suspension of 3 × 10^4^ Ad-MSCs in 200 μL of SCM. Cellularized patches were incubated at 37°C for at least seven days in a humidified atmosphere containing 5% CO_2_. Concomitantly, control patches with 200 μL of medium without cells were incubated under the same conditions. The medium was changed once. The decellularization procedure for the SF patches was performed by removing the culture medium, washing the patches with sterile distilled H_2_O at 4°C and incubating them with distilled MilliQ water for 30 minutes at 4°C. The D-Ad-MSCs-SF patches were then transferred into a new well and stored under different conditions: in distilled water at 4°C, under dry conditions at 4°C, frozen in distilled water at -20°C, or frozen under dry conditions at -20°C. D-Ad-MSCs-SF patches stored for more than seven days were not used *in vivo*.

### Conformational analysis of untreated and D-Ad-MSCs-SF patches

Conformational analysis was performed on untreated and decellularized patches to test whether sterilization, decellularization, and different storing conditions could affect the SF structure.

Fourier transform-infrared (FTIR) spectroscopy measurements were performed on a NEXUS (Thermo Nicolet) FTIR spectrometer employing an attenuated total reflectance (ATR) accessory model, Smart Performer. All spectra were obtained with a ZnSe crystal cell as the internal reflection element. Spectra were recorded in the 400 to 4,000 cm^-1^ wave number range by accumulating 64 scans at a resolution of 4 cm^-1^, subjected to data smoothing (15 points with the Savitzky-Golay method) and normalized to the 1,452 cm^-1^ peak before any data processing. Each spectrum was the average of at least three spectra measured in different areas of the sample. The crystallinity index was calculated as the intensity ratio between the two amide III components at 1260 cm^-1^ and 1230 cm^-1^ (C.I. = I_1260_/I_1230_) [28].

Differential scanning calorimetry (DSC) measurements were performed with a DSC Q200 instrument (TA Instruments, Waters, Milan, Italy). The scanned temperature ranged from room temperature to 400°C, at a heating rate of 10°C/minute. The analysis was carried out on samples of about 2 to 3 mg weight, in open aluminum pans of 40 μL volume, under N_2_ gas flux.

### Morphological analysis of SF, Ad-MSCs-SF and D-Ad-MSCs-SF patches

The morphology of SF, Ad-MSCs-SF and D-Ad-MSCs-SF patches was investigated by standard histology and by scanning electron microscopy (SEM) to evaluate the efficacy of cellularization and the presence of alterations in fibroin structure induced by sterilization, decellularization and different storing conditions. For histology, the samples were fixed in 10% neutral-buffered formalin, dehydrated and embedded in paraffin wax. Sections 5 μm thick were mounted on glass slides and stained with H&E. For SEM, patches were washed in 0.1 M pH 7.2 cacodylate buffer (CB), fixed with 2.5% glutaraldehyde (Sigma-Aldrich) in CB for two hours at 4°C and dehydrated in a graded series of ethanol up to absolute. Ad-MSCs-SF and D-Ad-MSCs-SF patches were placed on metal stubs, coated with gold to a thickness of 15 nm and viewed using a Philips XL30 scanning electron microscope (Center for Electron Microscopy - CUME, University of Perugia, Perugia, Italy).

### Immunophenotype

Ad-MSCs-SF patches were also subjected to immunofluorescence to confirm the maintenance of the Ad-MSC phenotype after cultivation on SF. Briefly, Ad-MSCs-SF cultured for seven days on SF patches were fixed for 30 minutes at room temperature in 4% PFA. The patches were permeabilized with 0.1% Triton X-100. The following primary antibodies were applied overnight at 4°C: mouse anti-human CD44 (1:500), CD45 (1:400), CD146 (1:400) and rabbit anti-human CD49d (1:500) (all purchased from Chemicon-Millipore, Temecula, California, USA). Cells were then incubated in Cy3-, and/or Cy2-conjugated secondary antibodies (Jackson ImmunoResearch, West Grove, PA, USA) at room temperature for 45 minutes and mounted with Fluorsave™ (Merck-Millipore, Nottingham, UK). The three-dimensional digital images were made using a Leica TCS SP2 AOBS (Leica Microsystems, Wetzlar, Germany) confocal laser scanning microscope.

### Surgical procedure: engraftment of Ad-MSCs-SF and D-Ad-MSCs-SF patches in diabetic (Leprdb/db) mice

Procedures involving animals were conducted in conformity with institutional guidelines in compliance with the Italian guidelines for animal care (DL 116/92), the European Community Council Directive (86/609/EEC) and the National Institutes of Health (NIH) Guide for the Care and Use of Laboratory Animals. The protocol for the use of laboratory animals was approved by the Italian Ministry of Health and by an internal ethics committee. All surgery was performed under chloral hydrate anesthesia and all efforts were made to minimize suffering.

Eight-week old Lepr *db/db* male mice (Charles River Laboratories, Calco, Lecco, Italy) were housed for at least one week in their home cages at a constant temperature. Mice were randomized into four groups of six each. Control no-graft mice received only saline and treated mice received SF, Ad-MSCs-SF and D-Ad-MSCs-SF patches, respectively.

Briefly, mice were anesthetized with chloral hydrate (4%, 400 mg/kg i.p) and one wound on each side of the mouse’s back midline extending through the *panniculus carnosus* was made with a sterile 5-mm punch biopsy tool (KLS Martin cod. 28-240-05-07 Germany). Patches were applied on the wounds and fixed to the skin with 30 μL of Hydrogel (ExtracelTM -X Maxi Hydrogel Kit, Glycosan BioSystems Inc. Alameda, CA, USA) ensuring prolonged adhesion to the wound. Mice were placed in individual home cages.

Mice were photographed postoperatively at three and ten days using a Canon high-resolution digital camera. Wound closure areas were measured as previously described [29]. Briefly, a metric ruler was placed adjacent to the wound limit and three individual photomicrographic measurements were taken for each mouse. The percentage of wound closure (contraction) was calculated with the formula: area at postoperative day 0 - area at postoperative day x/ area at postoperative day 0 × 100. All measurements were independently evaluated by two investigators blinded to the randomization.

Specimens of mouse skin treated with control SF, Ad-MSCs-SF and D-Ad-MSCs-SF patches were collected after transplants. Skin samples were fixed in 10% neutral buffered formalin, paraffin embedded, stained with H&E and examined histologically for the following main parameters: re-epithelialization, neovascularization and dermal fiber organization.

### Immunohistochemical analysis of Ad-MSCs-SF and D-Ad-MSCs-SF patches after transplantation

Tissue samples were collected 14 days after transplantation, fixed in 10% neutral buffered formalin and paraffin embedded. Immunohistochemistry was performed on 5 μm thick serial sections mounted on poly-l-lysine coated glass slides. After deparaffinization, endogenous peroxidase activity was quenched with 3% hydrogen peroxide in water. Sections were then microwaved for 15 minutes in 10 mM citric acid (pH 6.0) for antigen retrieval and treated with normal goat serum (Dako Corporation, Carpinteria, CA, USA) 1:10 for 30 minutes to prevent non-specific binding of primary antibodies. Subsequently, sections were incubated overnight at room temperature with the following primary antibodies: rabbit polyclonal anti-collagen IV antibody diluted 1:100 (Abcam, Cambridge, UK), mouse monoclonal anti-VEGF antibody diluted 1:100 (Abcam, Cambridge, UK), mouse monoclonal anti-human nuclei (HuNu) antibody diluted 1:50 (Merck Millipore, Billerica, MA, USA). The next day, after washing in PBS, the sections were incubated for 30 minutes at room temperature with the corresponding secondary biotin-conjugated antibody: goat anti-mouse immunoglobulin G (IgG) (for anti-HuNu and anti-VEGF) and goat anti-rabbit IgG (for anti-collagen IV antibody). Bound antibodies were revealed with avidin–biotin–peroxidase complex (Vector Elite kit, Vector Laboratories, Burlingame, CA, USA). The reaction was finally visualized by exposure to fresh diaminobenzidine chromogen substrate solution (Vector Laboratories). Negative controls without the primary antibody were run in parallel. Tissue observation was carried out using a light microscope (Nikon Eclipse E800, Nikon Corporation, Tokyo, Japan) connected to a digital camera (Dxm 1200 Nikon digital camera).

### Scratch test

The scratch assay was set up with an Ibidi Culture-Insert (Ibidi GmbH, Münich, Germany). Briefly, Ibidi Culture-Inserts were placed on the bottom of wells in a 24-multiwell plate coated with 0.2 mg/mL of collagen type I solution (Sigma-Aldrich). Human KCs, DFs and HUVECs (all purchased from Centro Substrati Cellulari, ISZLER, Brescia, Italy) were seeded to a final density of 5 × 10^3^ cell/cm^2^ onto an Ibidi Culture-Insert in SCM. The cells were maintained for 24 hours at 37°C and 5% CO_2_ to allow cell monolayer formation. Thereafter, the Ibidi Culture-Insert was removed, cells were gently washed with PBS, and 0.5 mL of SCM (without additional growth factors) was added. Next, transwells 8 μm polycarbonate membrane inserts filter (Corning Life Sciences) were placed on the well and then SF, Ad-MSCs-SF and D-Ad-MSCs-SF patches were located on the transwells and rapidly filled with 200 μL of SCM. After 24 hours or 48 hours, transwells were removed and cells were observed under a Zeiss Axiophot-2. To evaluate the reparative effects of SF patches, cells migrated into the inter-space were counted under microscopy at 20 × magnification in five random fields. Each migration test was run in triplicate.

At the end of the HUVECs scratch test, aliquots of conditioned medium (CM) were collected and, according to the manufacturer’s instructions, human FGF2, EGF (Mini ELISA Development kit purchased from Peprotech, Rocky Hill, New York, USA), TGFβ (Demeditec, Kiel-Wellsee, Germany) and VEGF (Ray Biotech, Inc, Georgia, USA) were quantified by ELISA in accordance with the standard guideline protocol supplied with the kits. The background value (<10%) of each growth factor contained in the SCM medium was assessed. Absorbance was measured at 450 nm or 405 nm with a microplate photometric reader DV990BV4 (GDV, Milan, Italy). The results were normalized with the CM obtained by the co-culture of HUVEC with SF patches and were expressed as mean ± SD of the secreted factor. The assay was repeated twice and each sample was run in triplicate.

### Quantitative reverse transcriptase–polymerase chain reaction

Reverse transcriptase–polymerase chain reaction (RT-PCR) was performed on Ad-MSCs cultured on plastic and on SF patches. For the analysis of mRNA levels, 500 ng of total RNA isolated using the RNeasy kit (Qiagen, Milan, Italy) was reverse-transcribed using an iScript cDNA Synthesis Kit (Bio-Rad Laboratories). Triplicate PCRs were carried out on an iCycler iQ Real Time PCR Detection System (Bio-Rad Laboratories, Milan, Italy). Relative gene expression was calculated by a comparative method (2^-ΔΔCt^) using GAPDH as a housekeeping gene. Primer sequences were designed using Primer3 software.

### Gene profile

Specimens of skin tissue were excised and analyzed for gene profile. Briefly, 500 ng of total RNA was used for each plate and was reverse-transcribed using RT^2^ First Strand Kit (SABioscience Corporation, Frederick, MD, USA), according to the manufacturer’s instructions. The RT^2^ Profiler™ PCR array for mouse wound healing (PAMM-121, SABioscience Corporation) was performed in triplicate for each sample to compare quantitative PCR (Q-PCR)–validated murine cDNA samples. Plates were run in Applied Biosystem AB 7300 real-time PCR, according to the manufacturer’s instructions. Relative gene expression was calculated using RT^2^ Profiler™ PCR array data analysis (SABioscience).

### Rat aortic ring assay

The rat aorta ring assay was also performed to investigate the angiogenic potential of Ad-MSCs-SF- and D-Ad-MSCs-SF-derived CMs during HUVECs scratch test. The aorta ring assay was performed as previously described [30]. Briefly, the dorsal aorta was excised from six-week-old Sprague–Dawley rats (Charles River Laboratories). Around 1 mm-thick rings were prepared. The rings were placed in a collagen solution prepared as described [31]. Around 40 μL of collagen solution was placed in each well and then one aortic ring/well was placed into the collagen solution. The plates were then incubated in a humidified incubator at 37°C for 30 minutes to obtain collagen jellification. At the end of incubation, the wells were filled with 500 μL of endothelial basal medium (EBM, LONZA, Walkersville, MD, USA) containing control medium, or SF, Ad-MSCs-SF and D-Ad-MSCs-SF derived CMs. The plates were incubated for 10 to 12 days. Quantification of angiogenesis was obtained by taking photographs with a Zeiss microscope at 10 × magnification every three days and by counting the number of microvessels arising from the aorta rings [29].

### Statistical analysis

The results are expressed as mean ± SD in replicate with n ≥3. One way analysis of variance (ANOVA) two-tailed test was utilized for all statistical analyses and performed with GraphPad Prism software, version 4.0. *P* values of less than 0.05 were considered to be significant.

## Results

### SF patches support adhesion and growth of Ad-MSCs

**Figure 1:**
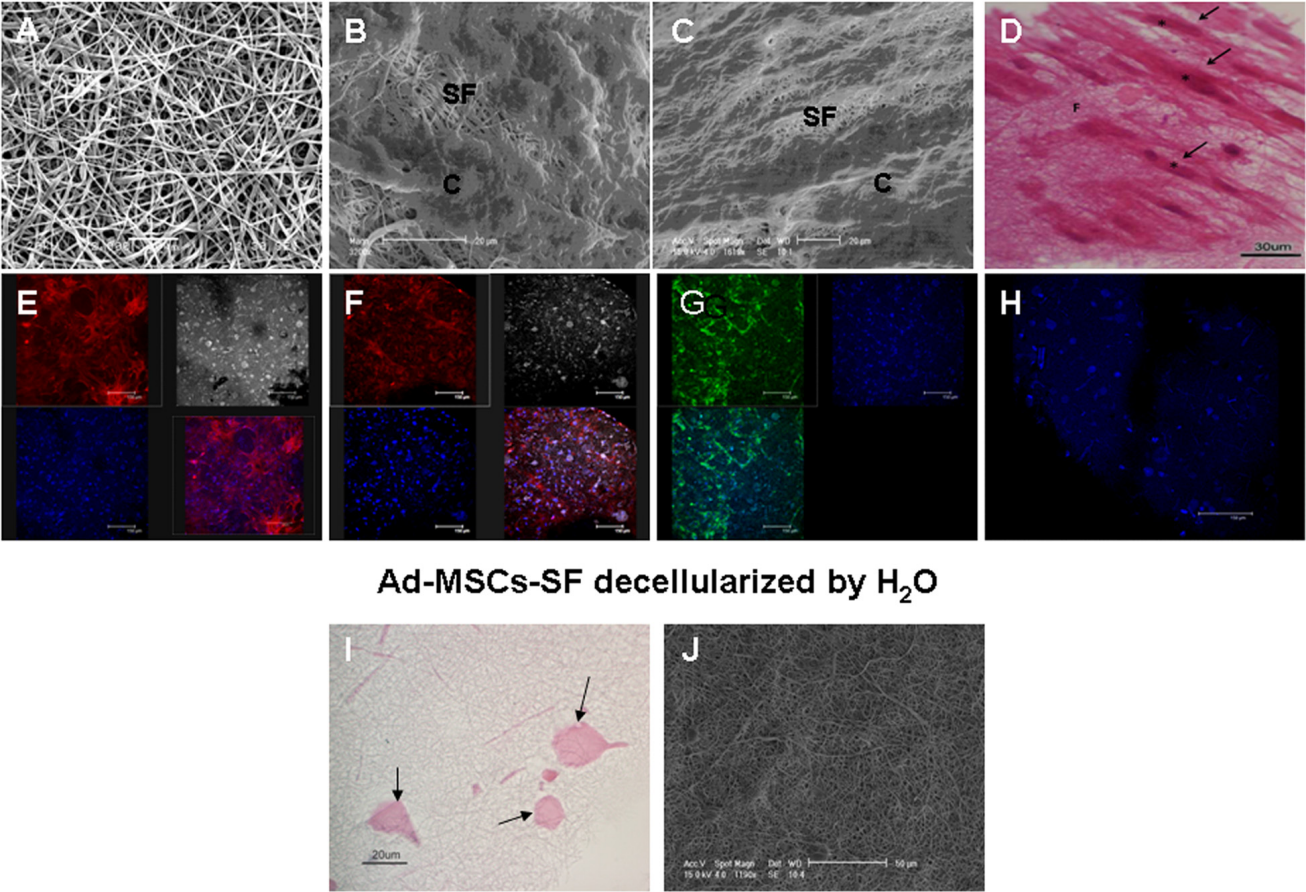
**Morphological appearance of SF patches seeded with Ad-MSCs before and after decellularization.** On SEM, untreated SF patches show their typical filamentous structure **(A)**. In **B** and **C,** SF patches seeded with Ad-MSCs after two and seven days of culture, respectively. Note that at seven days the Ad-MSCs formed an almost uniform monolayer on SF patches (Scale bar 20 μm). This result was also seen on light microscopy after H&E staining **(D)**; note that Ad-MSCs (arrows) have a spindle-shape morphology (asterisks indicate cell nuclei) (Scale bar 30 μm). After seven days of culture, Ad-MSCs-SF analyzed by confocal microscopy maintain the positive expression of mesenchymal markers CD146 **(E)** and CD44 **(F)** and of the integrin CD49d **(G)** and the negativity for the hematopoietic marker CD45 **(H)**. Incubation of Ad-MSCs-SF with distilled H_2_0 for one hour was effective in completely removing the cells from SF patches. H&E staining **(I)**: no cellular elements remain attached on SF patches after decellularization; the pink spots (arrows) represent fibroin aggregates (Scale bar 20 μm). SEM **(J)**: note the absence of cell debris (Scale bar 50 μm). In the figure: SF = silk fibroin; c = flattened Ad-MSCs. Ad-MSCs, adipose tissue derived mesenchymal stromal cells; SEM, scanning electron microscopy.

Cultured human Ad-MSCs showed a typical fibroblastic–like morphology (Additional file 1: Figure S1A). FC analysis of multiple surface epitopes showed that Ad-MSCs did not express (≤2%) hematopoietic cell markers CD14, CD34 and CD45, endothelial marker CD31 and immunological markers CD19 and HLA-DR. In contrast, Ad-MSCs highly expressed (≥90%) MSC markers, such as CD73, CD90 and CD105 (Additional file 1: Figure S1B). Moreover, when cultured under differentiation conditions, Ad-MSCs were able to differentiate towards adipocytes, osteocytes and chondrocytes (Additional file 1: Figure S1C-E). In order to investigate whether SF patches could support attachment, proliferation and maintenance of the phenotypic profile of Ad-MSCs, we seeded and cultured them on SF patches for seven days. We then analyzed their morphology by light microscopy and by SEM and evaluated their marker expression by immunofluorescence. SEM showed that the untreated SF patches appeared to be constituted of filamentous material consisting of nanofibers (Figure 1A). After the seeding, they were rapidly colonized by Ad-MSCs (Figure 1B) that formed a uniform and compact monolayer within seven days (Figure 1C). This result was also observed using light microscopy after H&E staining (Figure 1D). Confocal light microscopy further demonstrated that the Ad-MSCs cultured on SF patches maintained their phenotypic profile and were positive for the mesenchymal markers CD146 (Figure 1E) and CD44 (Figure 1F) and for the integrin CD49d (Figure 1G). Cells seeded on patches confirmed their negativity for the hematopoietic marker CD45 (Figure 1H). Moreover, when cultured on SF under differentiation conditions, the Ad-MSCs maintained the ability to differentiate towards the adipocytes, osteocytes and chondrocytes as demonstrated by Oil O Red and Alizarin Red staining and by aggrecan expression (Additional file 2: Figure S2A-C).

We next analyzed the morphology of SF patches after decellularization in order to validate our procedure. H&E staining (Figure 1I), as well as SEM analysis (Figure 1J), were performed on decellularized SF patches, demonstrating that the use of deionized MilliQ H_2_O for one hour was effective in removing all Ad-MSCs. The morphological analysis showed that after H_2_O treatment the cells were completely removed from the patches and no cell fragments remained entrapped within the SF patches. Furthermore, there was no apparent disruption of the architecture of the SF patches, as assessed by SEM analysis; in particular, the random arrangement typical of the SF fibers was maintained (Figure 1I-J).

FTIR and DSC were performed to assess the stability of untreated and D-Ad-MSCs-SF patches and to evaluate the influence of sterilization, decellularization, freezing (-20°C) and storing (4°C) under dry or wet conditions on intramolecular interactions.

FTIR and DSC analyses showed that the molecular and structural properties of SF patches were not affected by the decellularization process (Additional file 2: Figure S2D-E). The intrinsic crystalline structure of the SF patches did not change after the described treatments. Moreover, DSC analysis revealed that the presence and intensity of characteristic thermal transitions (glass transition, melting, degradation) recorded during heating may reflect typical structural features of the sample, SF alone, as demonstrated by the closely similar profiles reported in Additional file 2: Figure S2E. These results confirm that the SF patches were stable under the chemical and biological stressing conditions adopted in this study, in accordance with morphological data obtained by SEM analysis (see Figure 1I-J).

### Ad-MSC-SF and D–Ad-MSCs-SF improve skin healing in db/db diabetic mice

Diabetic *db/db* strain mice were used for *in vivo* tests. Each mouse underwent a 5-mm skin lesion with a sterile punch biopsy tool in a surgical procedure.

**Figure 2:**
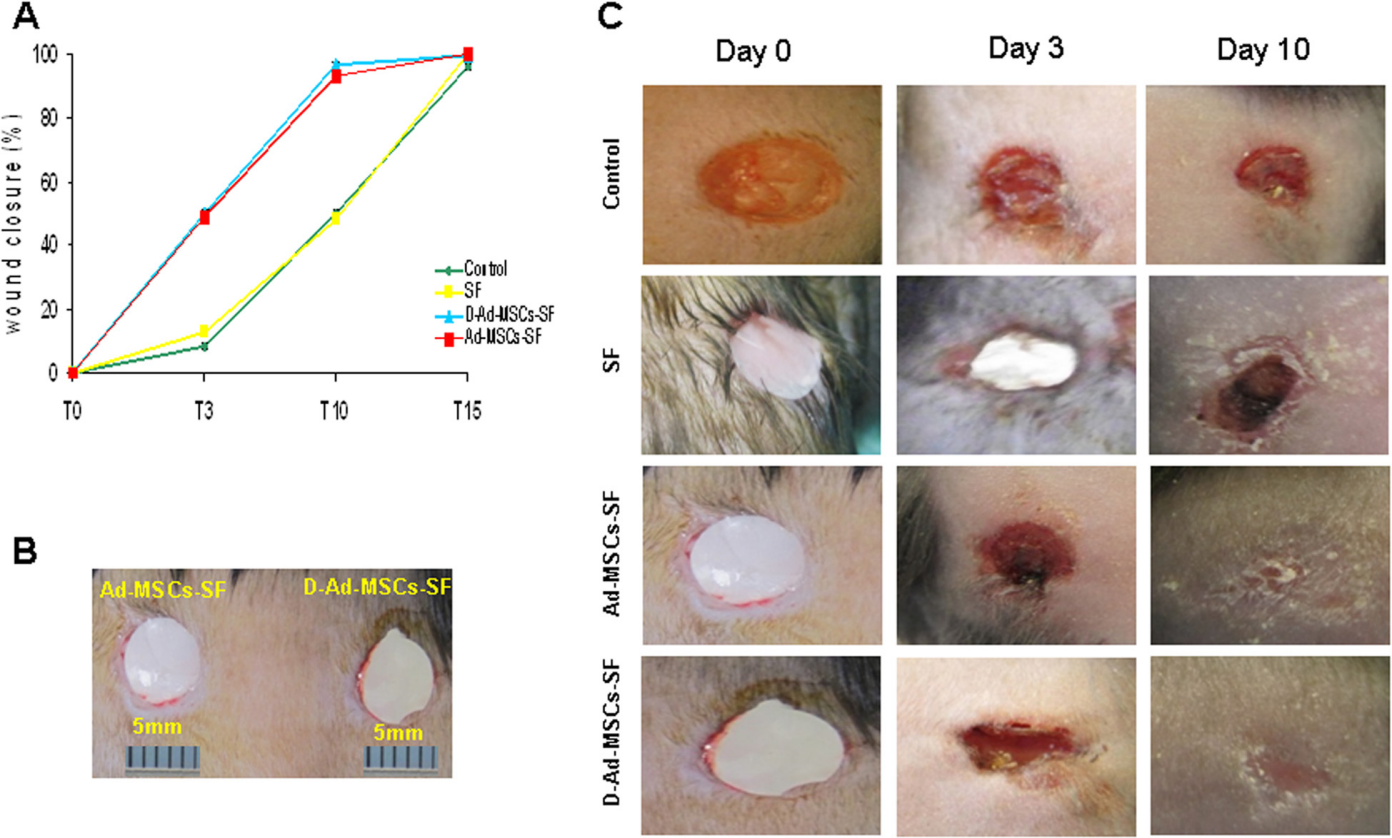
**Ad-MSCs-SF and D-Ad-MSCs-SF accelerate wound healing in diabetic mice.** Diabetic *db/db* strain mice were used for *in vivo* experiments. The different SF patches were applied on the wounds and fixed to the skin with 30 μL of hydrogel. **A)** shows the kinetics of wound closure. At each time the wound area was measured by using a metric ruler that was placed adjacent to the wound as shown in **B)**. Each point at the postoperative day 3, 10, 15 and 20 on the graph are the means ± SD of six mice. Note that on days 3 and 10, mice treated with Ad-MSCs-SF or with D-Ad-MSCs-SF have a significant increment of the wound closure area (*P <0.05, **P <0.01 versus no graft control). **C)** shows the photographs of the wounds taken at postoperative days 0, 3 and 10. Note that in mice treated with Ad-MSCs-SF and D-Ad-MSCs-SF, the skin wound was significantly reduced compared to either SF or to no graft mice controls. The ameliorative effect was evident even at day 3 after transplantation. In these mice the wounds were almost healed on day 10. Ad-MSCs-SF, silk fibroin patch cellularized with human adipose-derived mesenchymal stromal cells; D-Ad-MSCs-SF, silk fibroin patch after human adipose-derived mesenchymal stromal cells removal (decellularization); SF silk fibroin.

Wound closure was measured by planimetric analysis which revealed a strong increment of wound healing in mice treated with cellularized and, also, with decellularized SF scaffolds (Figure 2). In particular, wound closure at postoperative day 3 was 9.5% ± 0.5 in the control group, 11.5% ± 0.8 in the SF group, 49% ± 0.8 in the D-Ad-MSCs-SF group and 50% ± 2.4 in the Ad-MSCs-SF. Evaluations of the reduction of the wound area at postoperative day 10 were 50% ± 2.4 in the no graft group, 48% ± 1.9 in the SF group, 94% ± 2.9 in D-Ad-MSCs-SF group and 99.84% ± 1.9 in the Ad-MSCs-SF group (Figure 2A). The area of the wound was measured by using a metric ruler that was placed adjacent to the wound as shown in Figure 2B. In Figure 2C the photographs indicating the kinetics of wound healing in all groups are shown. Almost a complete epithelialization of the wound was reached around postoperative day 10 in mice treated with either cellularized or decellularized SF scaffold, while 15 to 17 days were necessary in control and SF grafted mice. The histological examination showed that the wound healing in control and in SF treated mice was delayed compared to Ad-MSCs-SF and D-Ad-MSCs-SF treated mice. However, at 14 days of treatment, those mice treated with Ad-MSCs-SF showed a higher degree of tissue organization not only in comparison with those treated only with the SF control, but even with those who received D-Ad-MSCs-SF patches. Indeed, epidermis and collagen fibers appeared well reorganized and even new hair follicles were present. In addition, a significant increment of microvascular density was observed (Additional file 3: Figure S3).

### Ad-MSCs-SF and D-Ad-MSCs-SF enhance the expression of genes involved in angiogenesis *in vivo*

**Figure 3:**
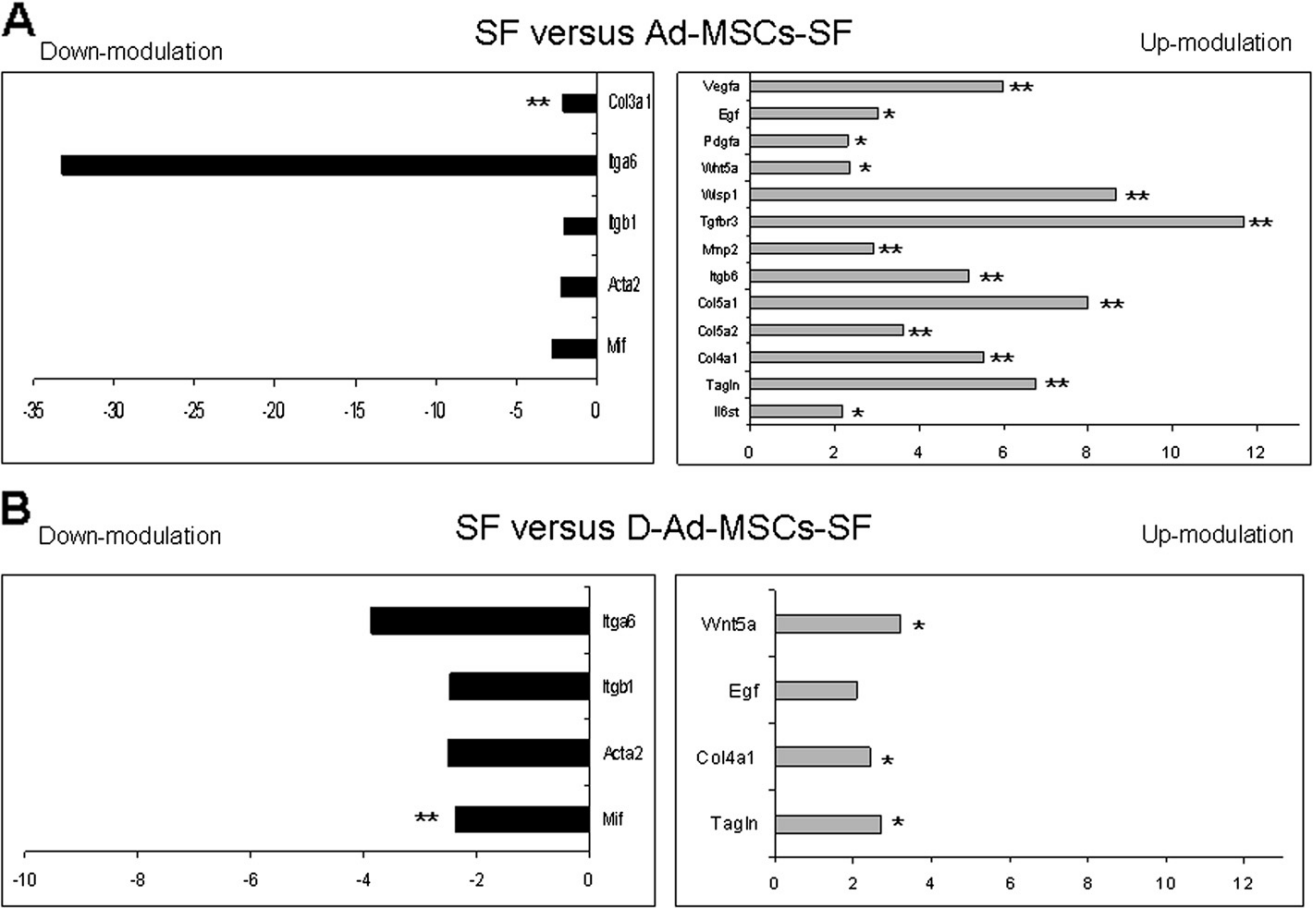
**Ad-MSCs-SF and D-Ad-MSCs-SF enhance genes involved in angiogenesis and ECM remodeling.** On day 7, specimens of skin tissue were excised and analyzed for gene profile. A significant increment of genes involved in angiogenesis and in tissue regeneration such as Vegfa, Egf and Pdgfa, Wnt5α, Wisp1 and Tgfβr3 and ECM remodeling (Mmp2, Itgβ6, Col5α1, Col5α2, Col4α1 and Tagln) were observed in Ad-MSCs-SF compared to SF treated mice **(A)**. Although less than in Ad-MSCs-SF, the RT^2^ gene profiler analysis of D-Ad-MSCs-SF treated mice revealed an increment of some genes involved in angiogenesis (Wnt5α, Egf), ECM deposition and remodeling (Col4α1, Tagln), as well as a down-modulation of genes involved in cytoskeleton organization (Itgα6, Itgβ1, Actα2). In general, in mice treated with D-Ad-MSCs-SF, inflammatory genes were not up-modulated and Mif was significantly down-regulated **(B)**. The RT^2^ Profiler™ array for mouse wound healing was performed in triplicate. Bars in the figure show the relative gene expression increment or decrement that was calculated using RT^2^ Profiler™ PCR Array Data Analysis. *P <0.05; **P <0.01 versus untreated SF patches. Ad-MSCs-SF, silk fibroin patch cellularized with human adipose-derived mesenchymal stromal cells; D-Ad-MSCs-SF, silk fibroin patch after human adipose-derived mesenchymal stromal cells removal (decellularization); ECM, extracellular matrix; SF, silk fibroin.

During *in vivo* wound regeneration, a sample of mice was sacrificed and tissues from the wound were removed in order to analyze gene expression. The RT^2^ gene profiler of Ad-MSCs-SF and D-Ad-MSCs-SF versus SF transplants is shown in Figure 3. In summary, mice treated with Ad-MSCs-SF showed a significant up-regulation of several genes involved in angiogenesis and in tissue regeneration, such as Vegfa, Egf, Pdgfa, Wnt5α, Wisp1 and Tgfβr3. In addition, genes involved in ECM deposition and remodeling such as Mmp2, Itgβ6, Col5α1, Col5α2, Col4α1 and Tagln were significantly increased. Conversely, we found a down-regulation of genes involved in collagen type 3 synthesis (Col3α1), adhesion and cytoskeleton organization (Itgα6, Itgβ1, Actα2). Genes involved in immunomodulation and inflammation, such as Il6st, were significantly increased, whereas macrophage migration inhibitor factor (Mif) was slightly down-regulated (Figure 3A). Although, less than in Ad-MSCs-SF, the RT^2^ gene profiler analysis of D-Ad-MSCs-SF treated mice revealed an increment of some genes involved in angiogenesis (Wnt5α, Egf), ECM deposition and remodeling (Col4α1, Tagln), as well as a down-modulation of genes involved in cytoskeleton organization (Itgα6, Itgβ1, Actα2). In general, in mice treated with D-Ad-MSCs-SF, inflammatory genes were not up-modulated and Mif was significantly down-regulated (Figure 3B).

Immunohistochemistry of the tissues from the mice that received Ad-MSCs-SF and D-Ad-MSCs-SF patches was also investigated for the angiogenic marker Vegf and for Col4α1 involved in basal membrane.

Expression of Col4α1 was observed in every sample. Basal membrane was continuously and sharply stained in Ad-MSCs-SF as well as in D-Ad-MSCs-SF demonstrating that the epidermal-dermal junction had been restored (Additional file 4: Figure S4A-C).

An average of 10 to 12 spindle-shaped Vegf positive cells per field (100× magnification) were observed in the dermal layer of Ad-MSCs-SF. Conversely, reactive cells in D-Ad-MSCs-SF treated samples were less numerous (2 to 4 per field, at 100× magnification) and were characterized by a less intense staining. A similar number of Vegf positive cells was detected in SF treated samples (Additional file 4: Figure S4D-F).

Immunohistochemical staining with anti-HuNu was also performed to demonstrate the ‘fate’ of human transplanted Ad-MSCs in host tissues. The anti-HuNu antibody reacted with some cells located in the dermal layer of Ad-MSCs-SF treated skin. An average of 3 to 4 positive cells per field (100× magnification) was detected. Anti-HuNu reactivity was never observed in D-Ad-MSCs-SF or SF treated skin (Additional file 4: Figure S4G-I).

### Ad-MSCs-SF and D-Ad-MSCs-SF improve migration of HUVECs

**Figure 4:**
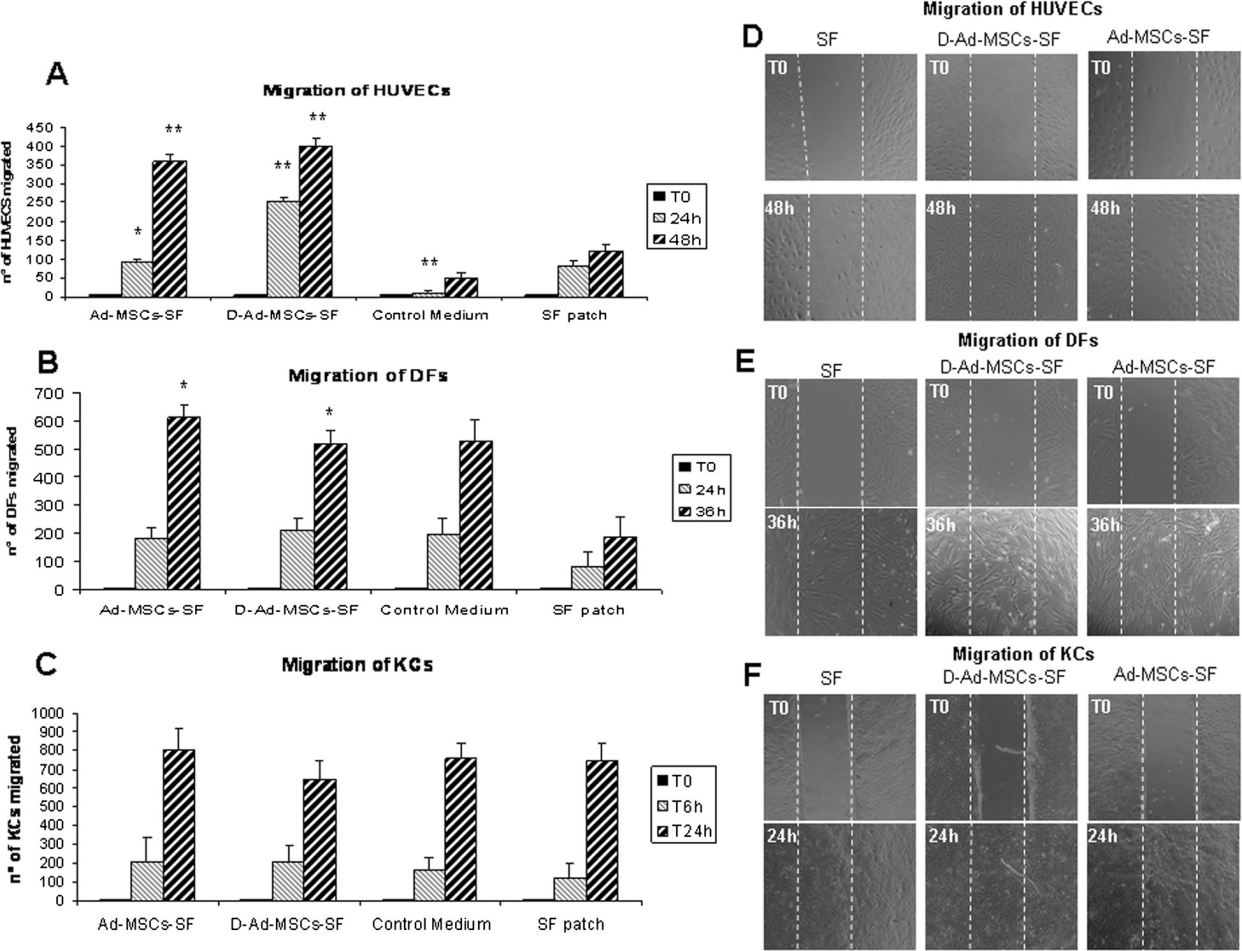
**Ad-MSCs-SF and D-Ad-MSCs-SF stimulate HUVECs migration by using the scratch test assay.** HUVECs, DFs and KCs were cultured in Ibidi Culture-Insert to confluence. Thereafter, the Ibidi Culture-Insert was removed and cells were co-cultured with control medium or in the presence of SF, Ad-MSCs-SF or D-Ad-MSCs-SF patches located in transwells (a scheme of the assay is also reported in Additional file 1: Figure S5). HUVECs **(A)**, DFs **(B)** and KCs **(C)** migrated into the inter-space were counted at different intervals of time under microscopy at 20× magnifications in five random fields. Bars in the figures are the means ± SD of three independent experiments. Each migration test was run in triplicate. *P <0.05; **P <0.01 versus untreated SF patches. During migration of HUVECs **(D)**, DFs **(E)** and KCs **(F)** photographs were taken to establish the time necessary for the cells to complete the coverage of scratch area. Note that HUVECs stimulated by D-Ad-MSCs-SF patches were the most efficient in completing the coverage of the scratch area that was completed in 48 hours. In the figure, the dotted white lines indicate the margin of the scratch. Ad-MSCs-SF, silk fibroin patch cellularized with human adipose-derived mesenchymal stromal cells; D-Ad-MSCs-SF, silk fibroin patch after human adipose-derived mesenchymal stromal cells removal (decellularization); DFs, dermal fibroblasts; HUVECs, human umbilical vein endothelial cells; KCs, keratinocytes; SF, silk fibroin.

Data from *in vivo* experiments showed that D-Ad-MSCs-SF possessed a significant capacity to improve wound healing in diabetic mice and that it was almost equal to that obtained with the Ad-MSCs-SF counterpart. Thus, SF patches could have the ability to work as a sponge, entrapping the molecules secreted by Ad-MSCs *in vitro* that are subsequently released *in vivo*. In order to verify that D-Ad-MSCs-SF release molecules that affect healing, we performed *in vitro* experiments on the migration of HUVECs, DFs and KCs by using the scratch test assay (a scheme of assay is reported in Additional file 5: Figure S5). As shown in Figure 4, migration of HUVECs was greatly enhanced under the influence of Ad-MSCs-SF and D-Ad-MSCs-SF patches compared to either untreated SF patches or trans-wells containing only the control medium (Figure 4A). Although less intensive, Ad-MSCs-SF and D-Ad-MSCs-SF induced a stimulation of DFs migration (Figure 4B) whereas no stimulation on migration of KCs was observed (Figure 4C). Interestingly, D-Ad-MSCs-SF patches were the most efficient in inducing a complete coverage of the scratch area by HUVECs, which was completed in 48 hours (Figure 4D). Instead, Ad-MSCs-SF and D-Ad-MSCs-SF were equally effective and needed 36 hours to complete the coverage of the scratch area by DFs (Figure 4E). KCs completed the coverage of the scratch area in 24 hours and no differences were observed among the various treatments (Figure 4F).

### Ad-MSCs-SF and D-Ad-MSCs-SF secrete angiogenic molecules in culture medium during endothelial cells migration

**Figure 5:**
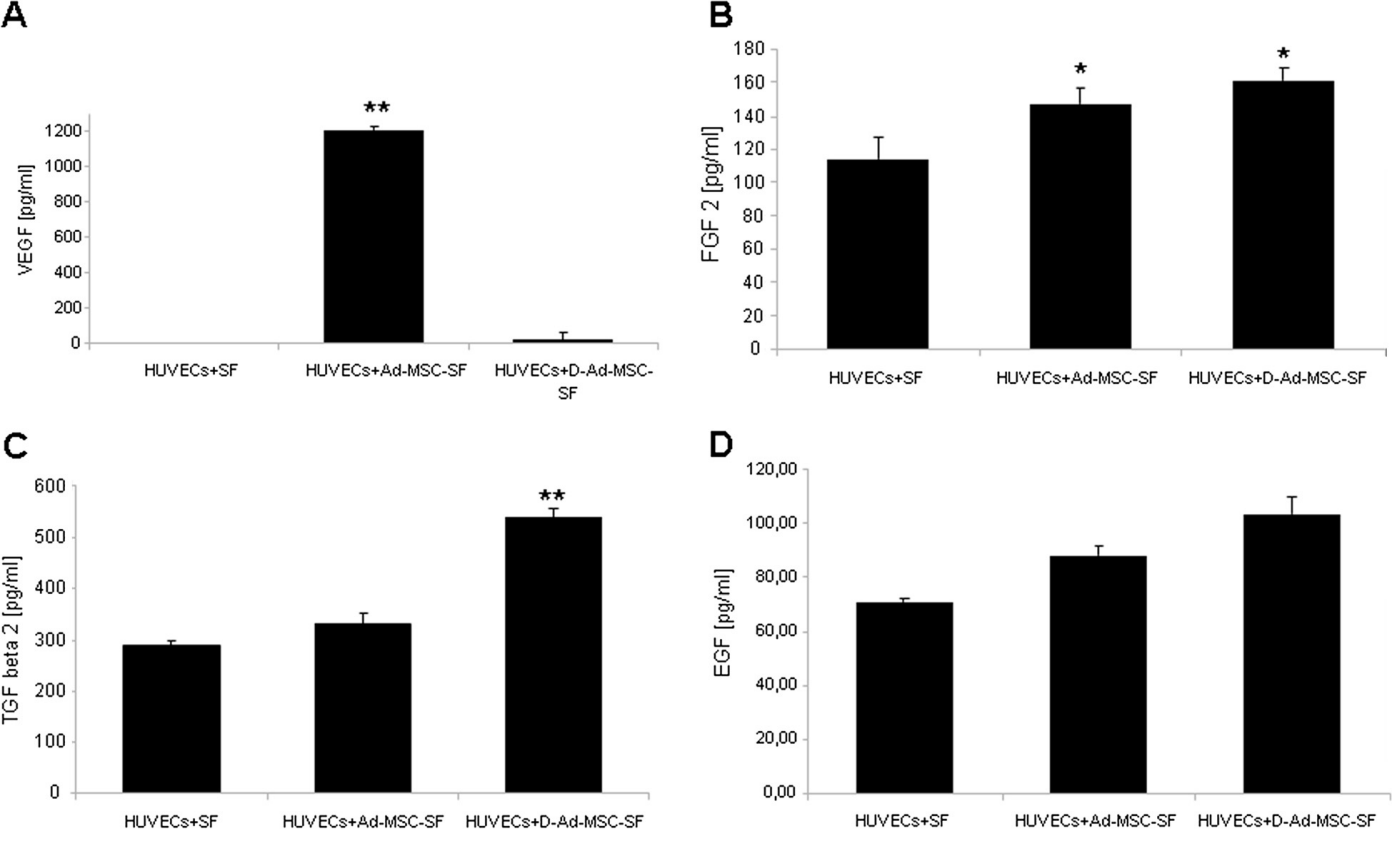
**Ad-MSCs-SF and D-Ad-MSCs-SF release angiogenic factors that stimulate migration of HUVECs.** At the end of the HUVECs scratch test, aliquots of CM were collected. The presence of human VEGF **(A)**, FGF2 **(B)**, TGFβ **(C)** and EGF **(D)** were quantified (pg/mL) by ELISA in accordance with the standard guideline protocol supplied with the kits. The background value (<10%) of each growth factor analyzed and contained in SCM not cultured with cells was subtracted. The results were normalized for the growth factor release by HUVEC co-cultured with untreated SF patches. Data are expressed as mean ± SD of the secreted factor. The assay was repeated twice and each sample was run in triplicate. *P <0.05; **P <0.01 versus HUVECs cultured in the presence of untreated SF patches. Ad-MSCs-SF, silk fibroin patch cellularized with human adipose-derived mesenchymal stromal cells; CM, conditioned medium; D-Ad-MSCs-SF, silk fibroin patch after human adipose-derived mesenchymal stromal cells removal (decellularization); EGF, epidermal growth factor; FGF2, fibroblast growth factor 2; HUVECs, human umbilical vein endothelial cells; SCM, stem cell medium; SF, silk fibroin; TGFβ, transforming growth factor β: VEGF, vascular endothelial growth factor.

In order to detect the angiogenic molecules released by Ad-MSCs-SF and D-Ad-MSCs-SF during migration of HUVECs, CM were collected and analyzed by ELISA. In particular, we evaluated the presence of VEGF, FGF2, TGFβ and EGF, all factors that have been shown to play an important role in wound angiogenesis [32,33] (Figure 5). The results were normalized for the growth factor release by HUVEC co-cultured with untreated SF patches. In summary, we found a significant increment of VEGF in Ad-MSCs-SF but not in D-Ad-MSCs-SF derived-CM (Figure 5A). Although at low concentration, FGF2 was statistically increased in CM of both Ad-MSCs-SF and D-Ad-MSCs-SF (Figure 5B) and the concentration of TGFβ was almost double in CM derived from D-Ad-MSCs-SF (Figure 5C). EGF was found in a very low concentration in CM of both Ad-MSCs-SF and D-Ad-MSCs-SF (Figure 5D). Additionally, we analyzed by RT-PCR the gene expression of VEGF, FGF2, TGFβ and EGF on Ad-MSCs cultured on plastic and on SF patches to ascertain whether SF patches may alter the expression of genes involved in the angiogenic stimulation. Compared to Ad-MSCs grown on plastic, Ad-MSCs-SF showed no significant differences in VEGF, FGF2 and TGFβ; only EGF expression was significantly down-modulated (Additional file 6: Figure S6).

### Ad-MSCs-SF and D-Ad-MSCs-SF stimulate outgrowth of microvessels in the aortic ring assay

**Figure 6:**
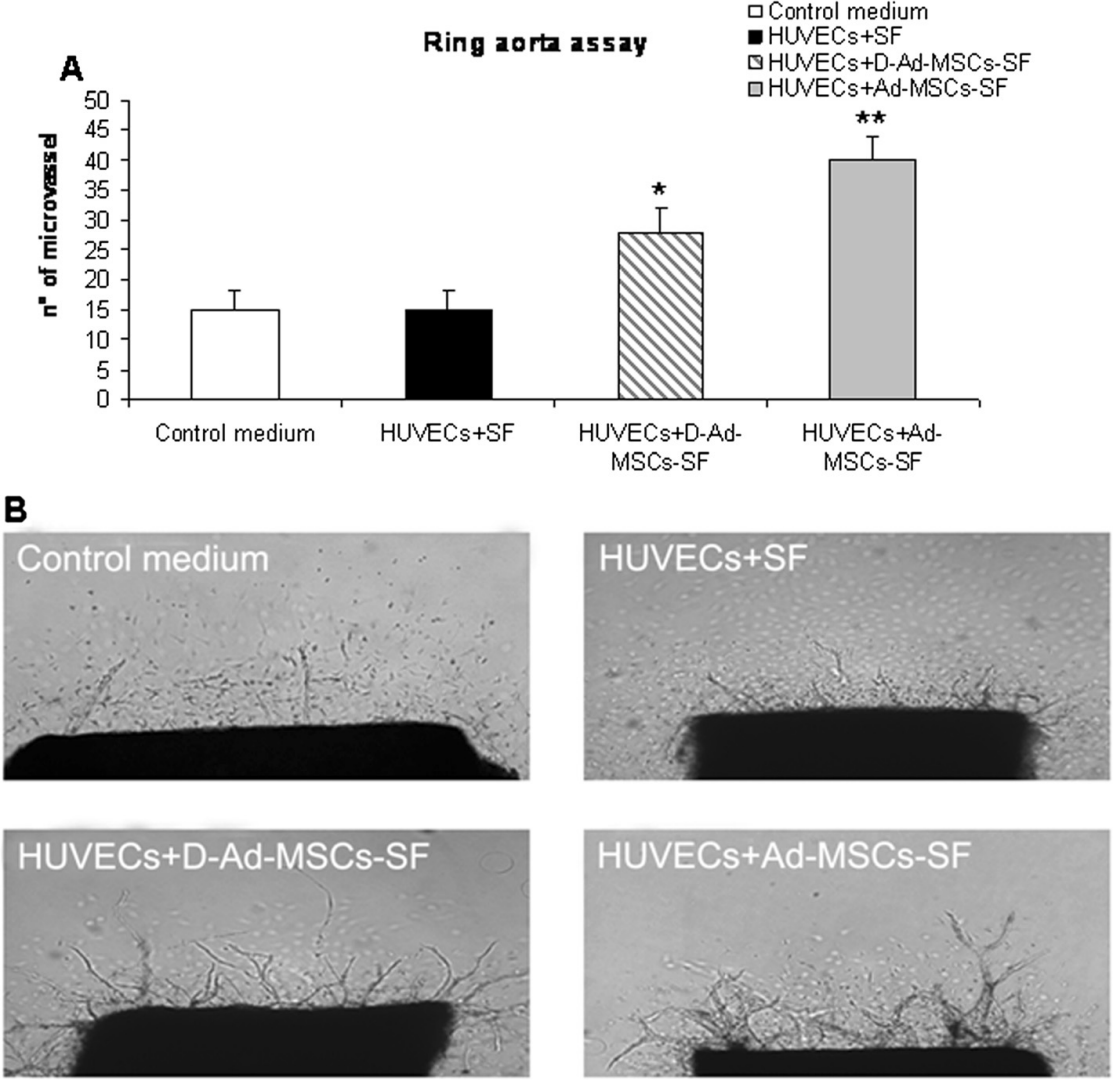
**Ad-MSCs-SF and D-Ad-MSCs-SF stimulate outgrowth of microvessels in the aorta ring assay.** Rat dorsal aorta was excised and around 1 mm-thick rings were prepared. The rings were placed into 40 uL of collagen solution and incubated at 37°C for 30 minutes to obtain collagen jellification. At the end of incubation of HUVECs with control medium or SF, Ad-MSCs-SF- and D-Ad-MSCs-SF-derived CM were added. In **A)** the quantification of neovessel outgrowth from aortic rings obtained by counting under an inverted microscope at 10× magnifications after 10 days of incubation is reported. Bars are means ± SD of number of neovessel formation. The assay was repeated twice and each sample was run in triplicate. *P <0.05; **P <0.01 versus rings cultured in the presence of CM derived from HUVECs treated with control SF patches. Photographs of capillary outgrowth from aorta rings were taken under the different treatment conditions **B)**. Ad-MSCs-SF, silk fibroin patch cellularized with human adipose-derived mesenchymal stromal cells; CM, conditioned medium; D-Ad-MSCs-SF, silk fibroin patch after human adipose-derived mesenchymal stromal cells removal (decellularization); HUVEC, human umbilical vein endothelial cells; SF, silk fibroin.

Since Ad-MSCs-SF and D-Ad-MSCs-SF secrete factors that stimulate HUVECs migration, we next evaluated the angiogenic potential of patches using the aortic ring assay to study the capacity of molecules to affect *ex vivo* formation of microvascular structures. To this end, CM was collected during the co-culture of Ad-MSCs-SF and D-Ad-MSCs-SF with HUVECs. As shown in Figure 6, treatment of aorta rings with Ad-MSCs-SF- and, to a minor extent, with D-Ad-MSCs-SF-derived CM improved capillary outgrowth. Maximal stimulation of angiogenesis was observed on day 7. At this time of observation, the number of capillaries in the presence of Ad-MSCs-SF-derived CM was 45 ± 5; it was 32 ± 8 in D-Ad-MSCs-SF, while in control medium and in SF-derived CM it was, respectively, 12 ± 6 and 15 ± 4 (Figure 6A). Figure 6B shows photographs of capillary outgrowth from aortic rings under the different treatment conditions.

## Discussion

SF patches are easy to handle, have a great mechanical strength, support cell adhesion and allow the storage and the release of growth factors produced by the cells [33-37]. For all these reasons, we have used SF patches cellularized with Ad-MSCs to test their capacity in improving wound healing in diabetic mice. In addition, we have examined the effects of D-Ad-MSCs-SF in order to determine if the presence of living cells at the transplantation site is essential to evoke reparative efficacy and if D-Ad-MSCs-SF may still retain biological efficacy in wound repair.

Previous data have shown that MSCs, either applied alone [32] or associated with SF-chitosan scaffold, demonstrated efficacy in accelerating wound healing in a murine model of soft tissue injury or in streptozotocin-induced diabetic rats [29,38]. More recently, O’Brien and colleagues demonstrated that topical administration of mesenchymal stem cells seeded in a collagen scaffold augments wound healing and increases angiogenesis in the diabetic rabbit ulcer [39].

We, here, significantly expanded these observations showing for the first time that not only Ad-MSCs-SF patches accelerate wound repair, but, more importantly, their decellularized SF counterpart worked as well. Moreover, different from other studies, we have used the *db/db* diabetic mice strain, an experimental animal model in which wound repair is significantly delayed, thus mimicking a pathological condition commonly observed in diabetic patients [22,40]. The rationale for using MSCs combined with SF patches to improve healing is based on previous experimental evidence showing that MSCs and SF may work synergistically [41-43]. According to these publications, SF scaffold mimicking a natural extracellular matrix, favors the adhesion and proliferation of MSCs and, when applied to a skin wound, may serve as a delivery substrate for MSCs [29]. On the other hand, the MSCs are considered cells with an active capacity to release angiogenic and trophic factors, critical to proper wound regeneration [32,33,44,45] or are able to differentiate into different cell lineages [46], including dermal cells [5,29,46]. In this regard, we clearly demonstrated that decellularized SF patches obtained by the simple procedure of using distilled H_2_O, that was sufficient to remove all the cells, maintained efficacy to accelerate healing. Thus, our data confirm that MSCs exert their wound healing efficacy mostly through a paracrine mechanism; therefore, the presence of live cells on the scaffold may not be needed [44,45]. Data from Altman *et al*., suggest a major trans-differentiation mechanism of human Ad-MSCs seeded on a SF-chitosan scaffold in enhancing wound repair [29]. However, they used immunodeficient mice, different from our *db/db* mice model which has an efficient immune system and, therefore, may rapidly eliminate the xenogenic human transplanted cells. Here, in the mice treated with Ad-MSCs-SF, some anti-HuNu positive cells were found; thus, in mice treated with live cells we cannot completely exclude a mechanism of transdifferentiation.

In our study, the paracrine mechanism of wound repair was also supported by *in vitro* experiments. In particular, by using the scratch test assay, we found that both Ad-MSCs-SF and D-Ad-MSCs-SF were able to significantly stimulate HUVECs and, to a lesser extent, the DFs, but not KCs migration. Interestingly, the analysis of CM during HUVECs migration, revealed that VEGF was highly produced only by Ad-MSCs-SF; in contrast, D-Ad-MSCs-SF release FGF2 and TFGβ but at very low levels (respectively, 160 and 540 pg/mL) probably insufficient *per se* to stimulate HUVECs migration. Therefore, while Ad-MSCs-SF may stimulate HUVECs migration through the release of VEGF, D-Ad-MSCs-SF appear to act through a more complex mechanism that may involve the synergic participation of different angiogenic factors (including perhaps FGF2 and TGFβ).

Concerning the capacity of SF to entrap molecules secreted by Ad-MSCs, we observed that D-Ad-MSCs-SF released FGF2 and TFGβ but not VEGF. Since Ad-MSCs-SF patches cultured in the absence of HUVECs did not reduce the expression of VEGF, FGF2 and TGFβ genes, we conclude that FGF2 and TGFβ may remain entrapped in the SF fibers while VEGF may not be entrapped and even if it remains trapped, it is not released. It is well known that a SF scaffold can be loaded with drugs or active growth factors [47,48]; however, whether SF scaffolds seeded with cells possess differential growth factors binding properties or a capacity to alter cell-releasing factors, remains to be further investigated. The significant down–modulation of Egf gene expression in Ad-MSCs when seeded on SF patches may suggest that, at least *in vitro*, SF may drive Ad-MSCs toward the production of growth factors (VEGF, TGFβ and FGF2) that affect MSCs rather than factors, such as EGF, that affect mostly neuroectodermal cells, such as KCs [49]. However, it is not clear if this is also true *in vivo*. Experiments using only bone marrow MSCs to treat wounds in diabetic rats showed an increase of the expression of Egf as well as mesenchymal growth factors [32].

In spite of the ability of SF to bind angiogenic factors produced by Ad-MSCs *in vitro*, the RT^2^ gene profile, performed in mice treated with cellularized and decellularized SF patches, seems to support our *in vitro* observations on angiogenesis. Indeed, albeit the RT^2^ gene profile of mice treated with Ad-MSCs-SF demonstrated a higher involvement of genes that control angiogenesis than those treated with D-Ad-MSCs-SF, both treatments seem to enhance the wound healing process through a mechanism that leads to stimulation of angiogenesis. Additionally, as the *in vivo* RT^2^ gene profile analysis also revealed that D-Ad-MSCs-SF treated mice may have a less inflammatory gene expression (in particular Mif and Il6st were significantly down-modulated), we cannot exclude the possibility that the absence of living xenogenic cells on the D-Ad-MSCs-SF patches may have less impact in stimulating inflammation, thus favoring skin regeneration [50].

## Conclusions

In conclusion, our results demonstrate that the combination of Ad-MSCs and SF patches is effective in accelerating wound repair in a model of diabetic mice that has been shown to have a significantly delayed capacity of wound healing [22]. More importantly, our study demonstrates for the first time that an almost equivalent success in wound repair can be achieved by using decellularized SF patches. Both treatments improve healing through a similar mechanism that mainly involves the release of angiogenic and collagen deposition stimulating molecules. The removal of the cells from SF patches may have significant and practical advantages in decreasing the risk of transferring genetically mutated cells and in reducing the possibility of stimulating the immune system. Moreover, decellularized patches could be prepared and stored until their use. Further studies will be performed on SF patches electrospinning with growth factors released by cell culture. In our opinion, this study may represent an important step toward the successful treatment of ulcers in diabetic patients.

## Abbreviations

Ad-MSCs: human-derived adipose mesenchymal stromal cells; Ad-MSCs-SF: silk fibroin patch cellularized with human adipose-derived mesenchymal stromal cells; ADM: adipocyte differentiation medium; CB: cacodylate buffer; CDM: chondrogenic differentiation medium; CM: conditioned medium; D-Ad-MSCs-SF: silk fibroin patch after human adipose-derived mesenchymal stromal cells removal (decellularization); DFs: dermal fibroblasts; (D)MEM: (Dulbecco’s) modified Eagle’s medium; DSC: differential scanning calorimetry; ECM: extracellular matrix; EGF: epidermal growth factor; ELISA: enzyme-linked immunosorbent assay; FC: flow cytometry; FGF: fibroblast growth factor; FTIR: Fourier transform infrared spectroscopy; H&E: hematoxylin and eosin; HuNu: human nuclei; HUVECs: human umbilical vein endothelial cells; KCs: keratinocytes; MSCs: mesenchymal stromal cells; OCT: optimum cutting temperature; ODM: osteogenesis differentiation medium; PBS: phosphate-buffered saline; PFA: paraformaldehyde; RT-PCR: reverse transcriptase polymerase chain reaction; SCM: stem cells medium; SEM: scanning electron microscopy; SF: silk fibroin; TGFβ: transforming growth factor beta; VEGF: vascular endothelial growth factor.

## Competing interests

The authors declare that they have no financial competing interests and that decellularized electrospun nanofibers SF patches for treatment of skin lesions are under consideration for patent.

## Authors’ contributions

SEN: manuscript writing, collection and assembly of data, final approval of manuscript. LP: morphological data analysis and interpretation. MD: carried out the ELISA tests and molecular data. AF: histological and immunohistochemistry stainings. GI: collection and assembly of data. FA: *in vivo* silk fibroin patches transplantation. SC: collection and assembly of data. GB: collection and assembly of molecular data. VT: carried out aortic ring assay. VC: histological and immunohistochemistry stainings. AB: carried out the mesenchymal differentiation assay. AP: collection and assembly of flow cytometry data. GF: production of silk fibroin. AA: production of silk fibroin. PC: morphological data analysis and interpretation. RC: *in vivo* isolation of silk fibroin patches. GM: collection and assembly of data and help to draft the manuscript. GA: conception and design, manuscript writing. EAP: administrative support, final approval of manuscript. All authors read and approved the final manuscript.

## Supplementary Material

Additional file 1: Figure S1 Phenotypic characterization of Ad-MSCs. A) Images of cultured human Ad-MSCs at passages three to five were taken on sub-confluent culture of two different preparations of Ad-MSCs. Note the typical fibroblastic–like morphology of cells. B) FC analysis of multiple surface epitopes, showed that Ad-MSCs minimally expressed (≤2%) hematopoietic cell markers CD14, CD34 and CD45, endothelial marker CD31 and immunological markers CD19 and HLA-DR. In contrast, Ad-MSCs highly expressed (≥90%) MSC markers such as CD73, CD90 and CD105. C) After induction, Ad-MSCs exhibited adipogenic, osteogenic and chondrogenic potentials which were demonstrated by staining of lipid droplets, calcium nodules stained and aggrecan deposition with (A), Oil red-O (B) Alizarin Red and (C ) aggrecan, respectively. Click here for file

Additional file 2: Figure S2 Ad-MSCs differentiation potential on SF patches and structural analyses of untreated and decellularized SF patches. Ad-MSCs seeded on SF patches, under inductive conditions, differentiated towards adipocytes, osteocytes and chondrocytes as demonstrated by (A) Oil O Red, (B) Alizarin red stainings and (C) aggrecan positivity. (D) FTIR-ATR spectra of SF patches. (a) Untreated control sample (C.I. = 0.69). (b) SF patch sterilized with ethanol 70 vol% and exposed to UV light for one hour (C.I. = 0.67). (c) Decellularized SF patch stored in water at 4°C (C.I. = 0.69). (d) Decellularized SF patch stored under dry conditions at 4°C (C.I. = 0.69). (e) Decellularized SF patch frozen stored in water at -20°C (C.I. = 0.69). (f) Decellularized SF patch frozen stored under dry conditions at -20°C (C.I. = 0.69). (A I = amide I; A II = amide II; A III = amide III). The intrinsic crystalline structure of SF patches was not affected by any of the treatments carried out on them, from sterilization to decellularization, freezing and storing under dry or wet conditions at +4°C or -20°C, as demonstrated by the closely similar profiles and by the values of crystallinity. (E) DSC thermograms of SF patches. (a) Untreated control sample (C.I. = 0.69). (b) SF patch sterilized with ethanol 70 vol% and exposed to UV light for six hours. (c) Decellularized SF patch stored in water at 4°C. (d) Decellularized SF patch stored under dry conditions at 4°C. (e) Decellularized SF patch frozen stored in water at -20°C. (f) Decellularized SF patch frozen stored under dry conditions at -20°C. Sterilization caused a slight low-temperature broadening of the melting/degradation endotherm, but the main peak still remained at high temperature (284°C) and the β-sheet crystalline regions retained their thermal stability, as indicated by the FTIR results. Click here for file

Additional file 3: Figure S3 Histological analysis of skin wounds upon treatment with SF, Ad-MSCs-SF and D-Ad-MSCs-SF patches. On day 14 after treatments, some mice were sacrificed and wounds were investigated by histology. Control wounds treated with SF patches alone showed a dermis displaying important hypercellularity, scanty collagen fiber alignment and continuous epidermis with evident signs of dysplasia determined by the immature status (A, B). Wounds treated with D-Ad-MSCs-SF patches showed a more advanced epidermal organization and a dermis very rich in cells and microvessels (C, D). The wound treated with Ad-MSCs-SF showed the highest degree of tissue organization (E, F); the multilayer structure of epidermis was formed, the dermis still showed hypercellularity with the presence of numerous neoformed small vessels. It was also possible to observe early pilo-sebaceous units (arrowheads). In B, D and F are shown, at higher magnifications, the skin of mice treated with SF, D-Ad-MSCs-SF and Ad-MSCs-SF patches, respectively. In the figure, wound edges are indicated by arrows; e = epidermis; d = dermis. Click here for file

Additional file 4: Figure S4 Wound healing process in mouse tissue by Ad-MSCs-SF and D-Ad-MSCs-SF. In mouse tissues that received SF, Ad-MSCs-SF and D-Ad-MSCs-SF patches, Col4α1 (A,B,C) and Vegf (D,E,F) were investigated by immunohistochemistry. Expression of Col4α1was observed in every sample. Basal membrane was continuously and sharply stained in Ad-MSCs-SF as well as in D-Ad-MSCs-SF demonstrating that the epidermal-dermal junction had been restored. An average of 10 to 12 spindle shaped Vegf positive cells per field (100× magnification) were observed in the dermal layer of Ad-MSCs-SF. Conversely, reactive cells in D-Ad-MSCs-SF treated samples were less numerous (two to four per field, at 100× magnification) and were characterized by a less intense staining. A similar number of Vegf positive cells was detected in SF treated samples. Immunohistochemical staining with anti-HuNu was additionally performed to demonstrate the ‘fate’ of human transplanted Ad-MSCs in host tissues. The anti-HuNu antibody reacted with some cells located in the dermal layer of Ad-MSCs-SF treated skin. An average of three to four positive cells per field (100× magnification) was detected. Anti-HuNu reactivity was never observed in D-Ad-MSCs-SF and SF treated skin (G,H,I). Click here for file

Additional file 5: Figure S5 Scheme of the scratch test assay to evaluate SF, D-Ad-MSCs-SF and Ad-MSCs-SF activity on cell migration. As shown in the figure, the scratch assay was set up with an Ibidi Culture-Insert placed on the bottom of wells in a 24-multiwell plate (A). Human KCs, DFs and HUVECs seeded into Ibidi Culture-Insert in SCM allow cell monolayer formation. Thereafter, the Ibidi Culture-Insert was removed and 0.5 mL of SCM was added. Next, transwells 8-μm Polycarbonate Membrane Inserts filter were placed on the well and then SF (B), D-Ad-MSCs-SF (C) and Ad-MSCs-SF (D) were placed into transwells and rapidly filled with 200 μL of SCM. For HUVECs, transwells were removed after 24 hours or 48 hours and cells were observed under a Zeiss Axiophot-2 microscope. Cells migrated into the inter-space were counted under microscopy at 20× magnifications in five random fields. Each migration test was run in triplicate. Click here for file

Additional file 6: Figure S6 Angiogenic genes expression of Ad-MSCs seeded on SF patches. RT-PCR was performed to evaluate the gene expression of VEGF, FGF2, TGFβ and EGF on Ad-MSCs cultured on plastic and on SF patches. On the ordinate axis is reported the fold of changes of gene expression of Ad-MSCs on SF patches versus control Ad-MSCs cultured on plastic. The control of gene expression values was considered equal to 1. Relative gene expression was calculated by a comparative method (2^-ΔΔCt^) using GAPDH as a housekeeping gene. Polymerase chain reactions were carried out in triplicate. Note that in comparing Ad-MSCs grown on plastic versus Ad-MSCs-SF, no significant differences in VEGF, FGF2 and TGFβ were seen; only EGF expression was significantly down-modulated. *P <0.05 versus SF patches. Click here for file
