# Supplementary figures and images for: Decellularized silk fibroin scaffold primed with adipose mesenchymal stromal cells improves wound healing in diabetic mice
Source: Stem Cell Res Ther. 2014 Jan 14;5(1):7. doi: 10.1186/scrt396 (PMC4055150; doi:10.1186/scrt396)

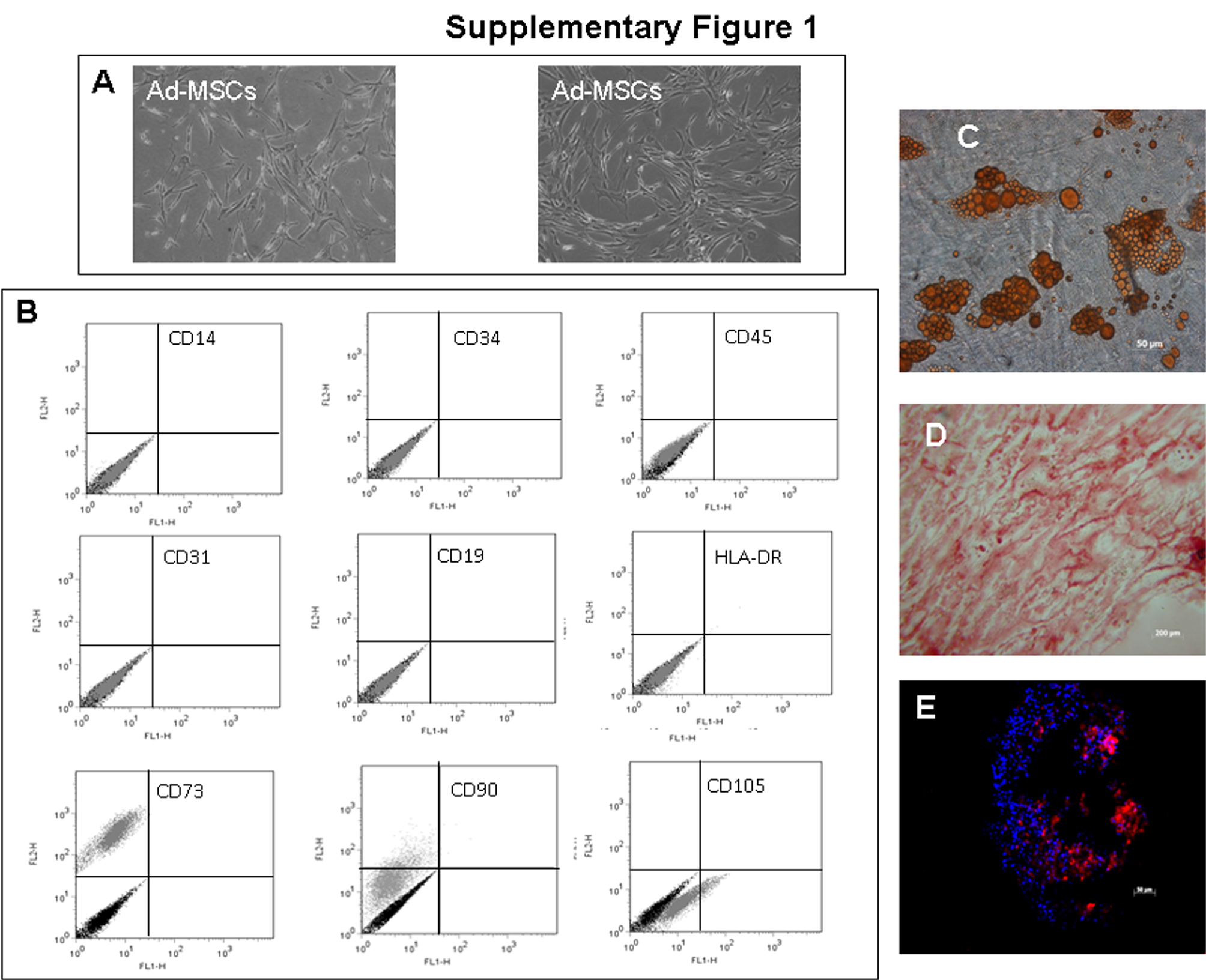

Supplement: Additional file 1: Figure S1 — Phenotypic characterization of Ad-MSCs. A) Images of cultured human Ad-MSCs at passages three to five were taken on sub-confluent culture of two different preparations of Ad-MSCs. Note the typical fibroblastic–like morphology of cells. B) FC analysis of multiple surface epitopes, showed that Ad-MSCs minimally expressed (≤2%) hematopoietic cell markers CD14, CD34 and CD45, endothelial marker CD31 and immunological markers CD19 and HLA-DR. In contrast, Ad-MSCs highly expressed (≥90%) MSC markers such as CD73, CD90 and CD105. C) After induction, Ad-MSCs exhibited adipogenic, osteogenic and chondrogenic potentials which were demonstrated by staining of lipid droplets, calcium nodules stained and aggrecan deposition with (A), Oil red-O (B) Alizarin Red and (C ) aggrecan, respectively. [file scrt396-S1.tiff]

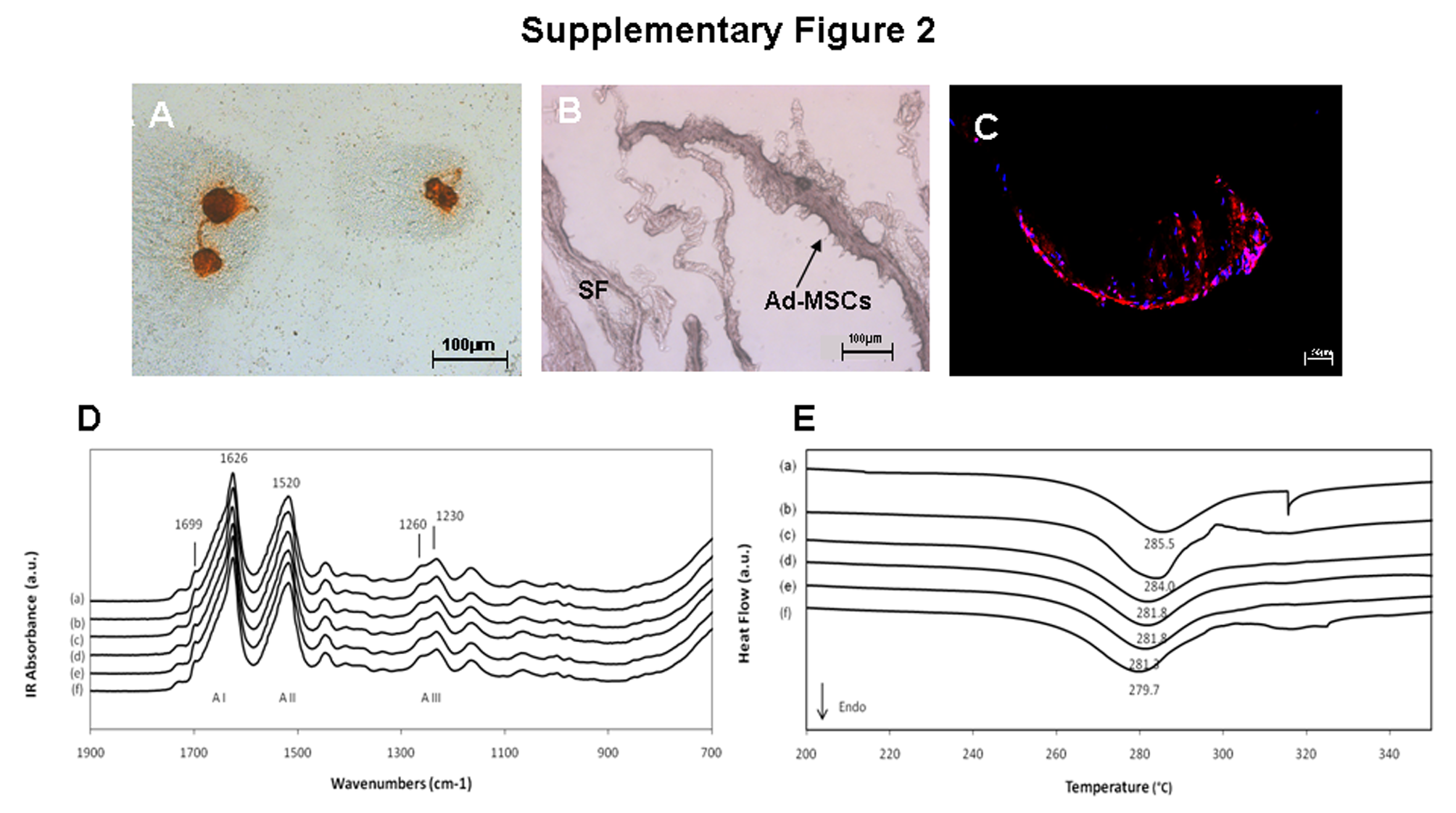

Supplement: Additional file 2: Figure S2 — Ad-MSCs differentiation potential on SF patches and structural analyses of untreated and decellularized SF patches. Ad-MSCs seeded on SF patches, under inductive conditions, differentiated towards adipocytes, osteocytes and chondrocytes as demonstrated by (A) Oil O Red, (B) Alizarin red stainings and (C) aggrecan positivity. (D) FTIR-ATR spectra of SF patches. (a) Untreated control sample (C.I. = 0.69). (b) SF patch sterilized with ethanol 70 vol% and exposed to UV light for one hour (C.I. = 0.67). (c) Decellularized SF patch stored in water at 4°C (C.I. = 0.69). (d) Decellularized SF patch stored under dry conditions at 4°C (C.I. = 0.69). (e) Decellularized SF patch frozen stored in water at -20°C (C.I. = 0.69). (f) Decellularized SF patch frozen stored under dry conditions at -20°C (C.I. = 0.69). (A I = amide I; A II = amide II; A III = amide III). The intrinsic crystalline structure of SF patches was not affected by any of the treatments carried out on them, from sterilization to decellularization, freezing and storing under dry or wet conditions at +4°C or -20°C, as demonstrated by the closely similar profiles and by the values of crystallinity. (E) DSC thermograms of SF patches. (a) Untreated control sample (C.I. = 0.69). (b) SF patch sterilized with ethanol 70 vol% and exposed to UV light for six hours. (c) Decellularized SF patch stored in water at 4°C. (d) Decellularized SF patch stored under dry conditions at 4°C. (e) Decellularized SF patch frozen stored in water at -20°C. (f) Decellularized SF patch frozen stored under dry conditions at -20°C. Sterilization caused a slight low-temperature broadening of the melting/degradation endotherm, but the main peak still remained at high temperature (284°C) and the β-sheet crystalline regions retained their thermal stability, as indicated by the FTIR results. [file scrt396-S2.tiff]

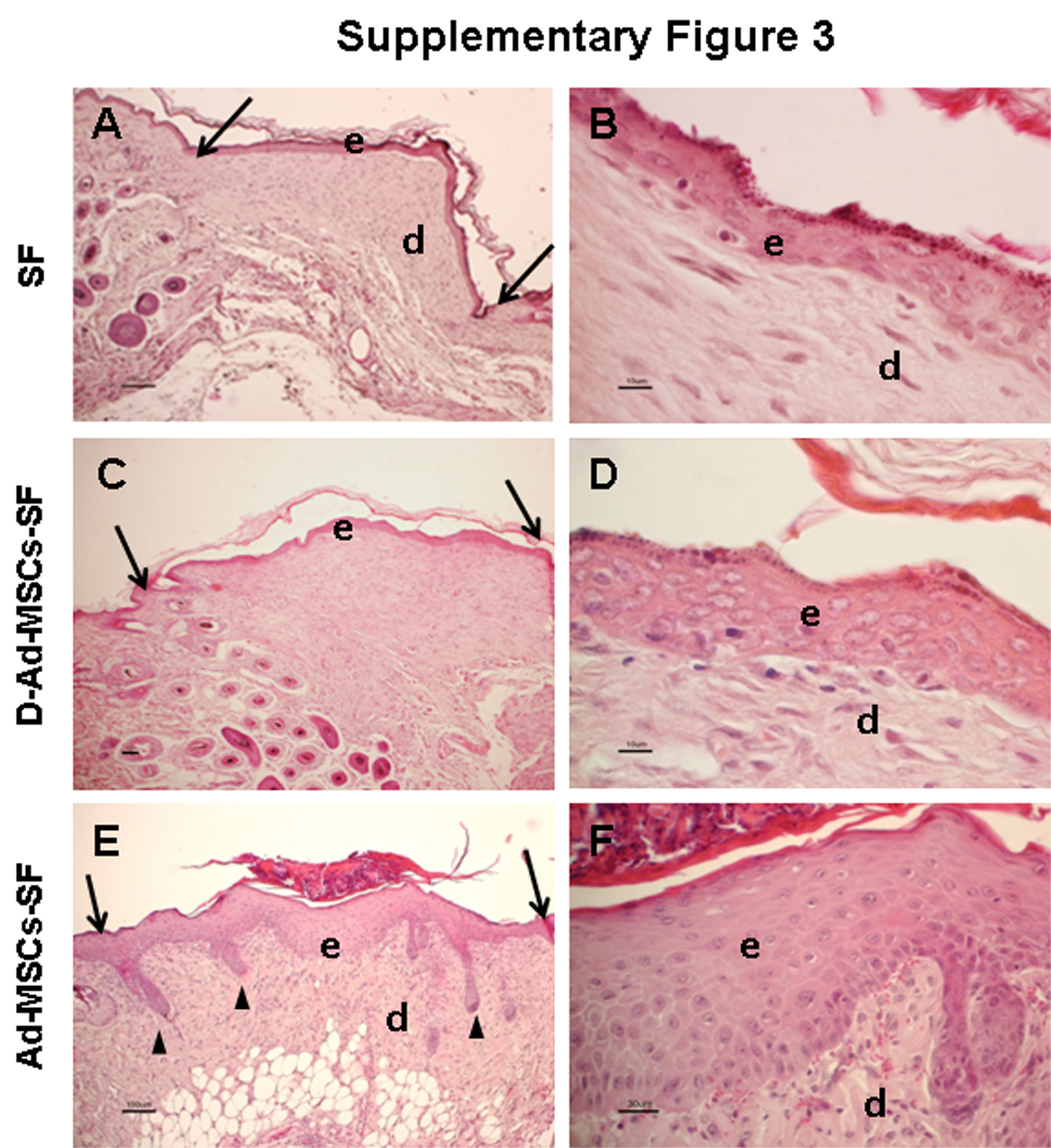

Supplement: Additional file 3: Figure S3 — Histological analysis of skin wounds upon treatment with SF, Ad-MSCs-SF and D-Ad-MSCs-SF patches. On day 14 after treatments, some mice were sacrificed and wounds were investigated by histology. Control wounds treated with SF patches alone showed a dermis displaying important hypercellularity, scanty collagen fiber alignment and continuous epidermis with evident signs of dysplasia determined by the immature status (A, B). Wounds treated with D-Ad-MSCs-SF patches showed a more advanced epidermal organization and a dermis very rich in cells and microvessels (C, D). The wound treated with Ad-MSCs-SF showed the highest degree of tissue organization (E, F); the multilayer structure of epidermis was formed, the dermis still showed hypercellularity with the presence of numerous neoformed small vessels. It was also possible to observe early pilo-sebaceous units (arrowheads). In B, D and F are shown, at higher magnifications, the skin of mice treated with SF, D-Ad-MSCs-SF and Ad-MSCs-SF patches, respectively. In the figure, wound edges are indicated by arrows; e = epidermis; d = dermis. [file scrt396-S3.tiff]

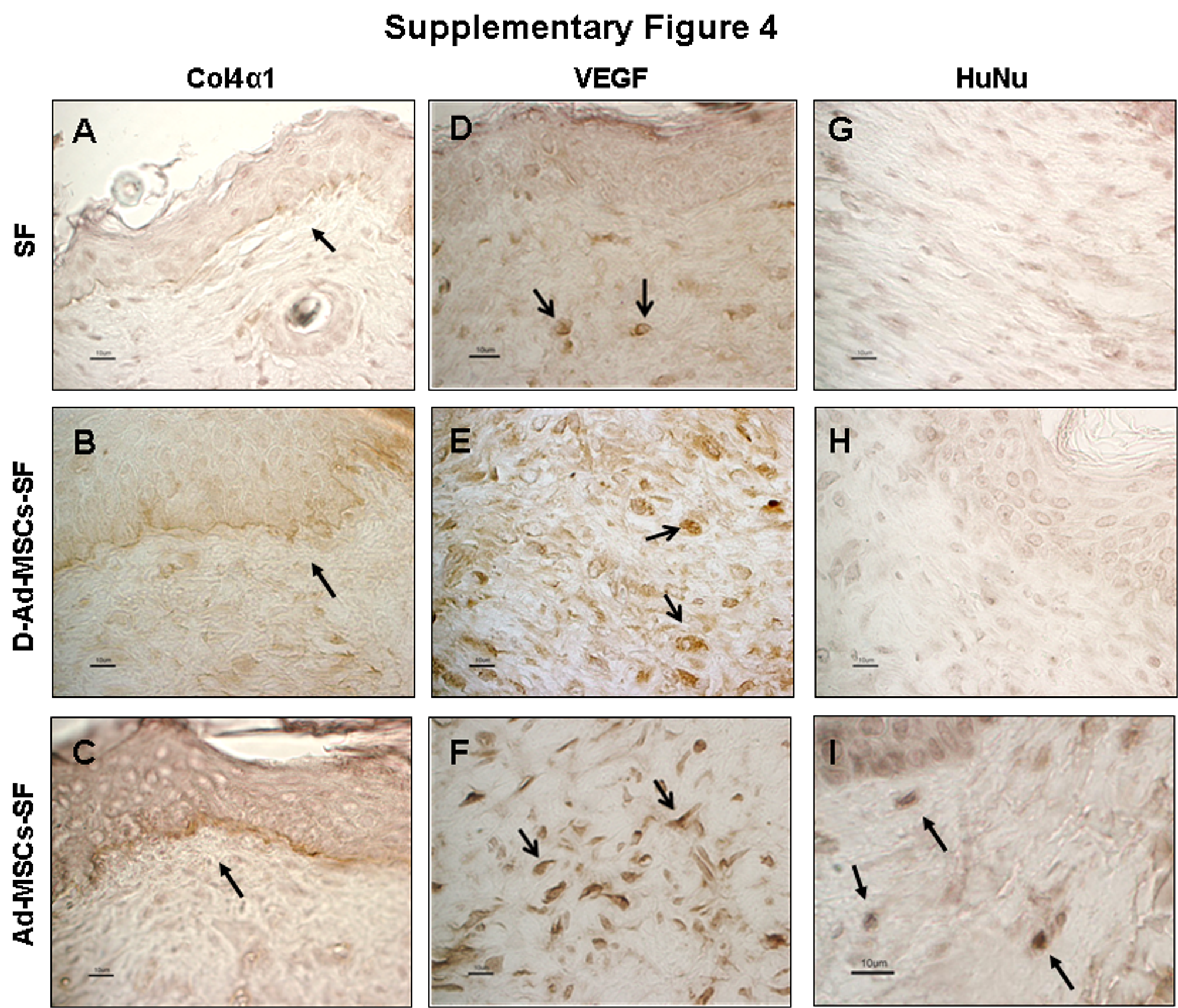

Supplement: Additional file 4: Figure S4 — Wound healing process in mouse tissue by Ad-MSCs-SF and D-Ad-MSCs-SF. In mouse tissues that received SF, Ad-MSCs-SF and D-Ad-MSCs-SF patches, Col4α1 (A,B,C) and Vegf (D,E,F) were investigated by immunohistochemistry. Expression of Col4α1was observed in every sample. Basal membrane was continuously and sharply stained in Ad-MSCs-SF as well as in D-Ad-MSCs-SF demonstrating that the epidermal-dermal junction had been restored. An average of 10 to 12 spindle shaped Vegf positive cells per field (100× magnification) were observed in the dermal layer of Ad-MSCs-SF. Conversely, reactive cells in D-Ad-MSCs-SF treated samples were less numerous (two to four per field, at 100× magnification) and were characterized by a less intense staining. A similar number of Vegf positive cells was detected in SF treated samples. Immunohistochemical staining with anti-HuNu was additionally performed to demonstrate the ‘fate’ of human transplanted Ad-MSCs in host tissues. The anti-HuNu antibody reacted with some cells located in the dermal layer of Ad-MSCs-SF treated skin. An average of three to four positive cells per field (100× magnification) was detected. Anti-HuNu reactivity was never observed in D-Ad-MSCs-SF and SF treated skin (G,H,I). [file scrt396-S4.tiff]

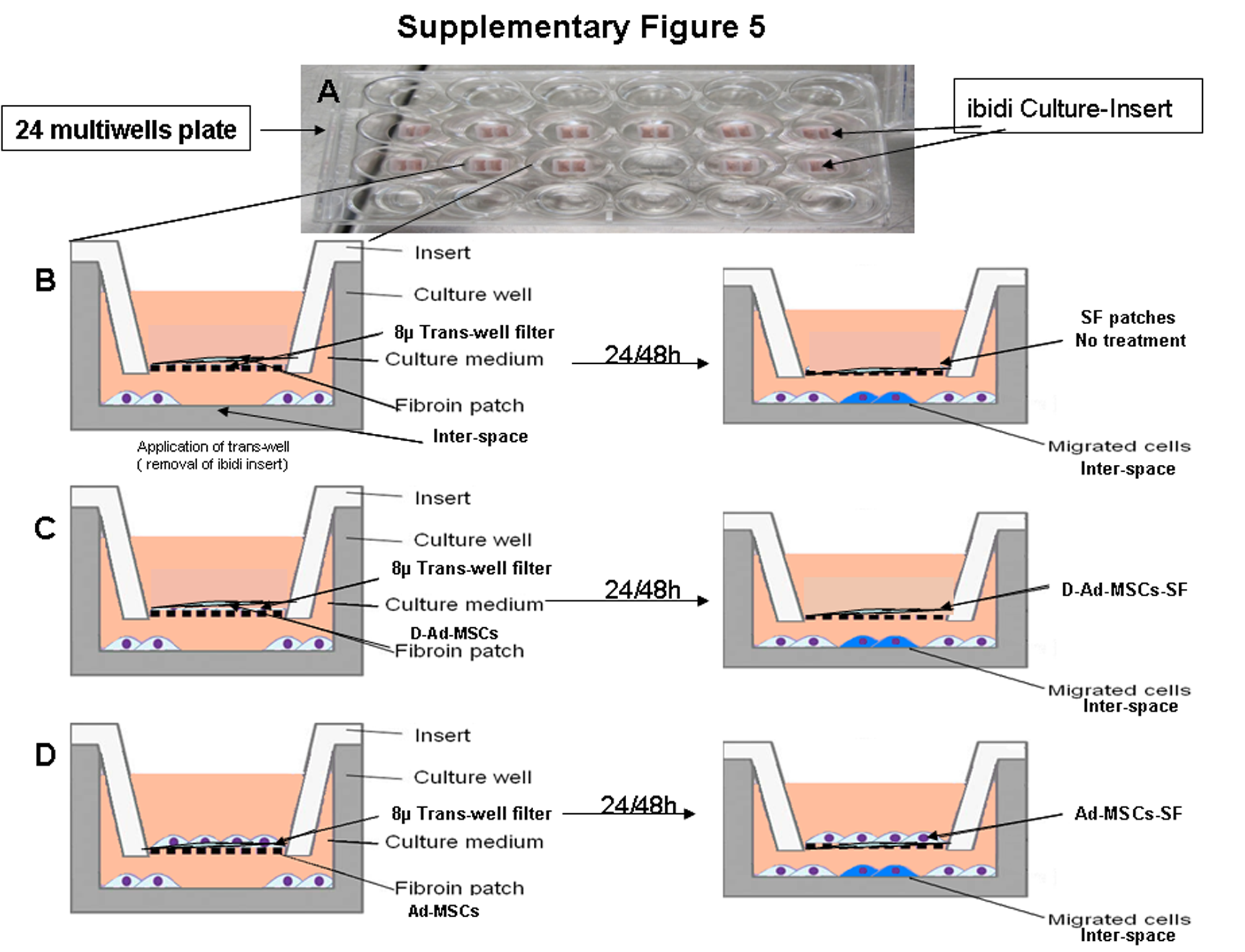

Supplement: Additional file 5: Figure S5 — Scheme of the scratch test assay to evaluate SF, D-Ad-MSCs-SF and Ad-MSCs-SF activity on cell migration. As shown in the figure, the scratch assay was set up with an Ibidi Culture-Insert placed on the bottom of wells in a 24-multiwell plate (A). Human KCs, DFs and HUVECs seeded into Ibidi Culture-Insert in SCM allow cell monolayer formation. Thereafter, the Ibidi Culture-Insert was removed and 0.5 mL of SCM was added. Next, transwells 8-μm Polycarbonate Membrane Inserts filter were placed on the well and then SF (B), D-Ad-MSCs-SF (C) and Ad-MSCs-SF (D) were placed into transwells and rapidly filled with 200 μL of SCM. For HUVECs, transwells were removed after 24 hours or 48 hours and cells were observed under a Zeiss Axiophot-2 microscope. Cells migrated into the inter-space were counted under microscopy at 20× magnifications in five random fields. Each migration test was run in triplicate. [file scrt396-S5.tiff]

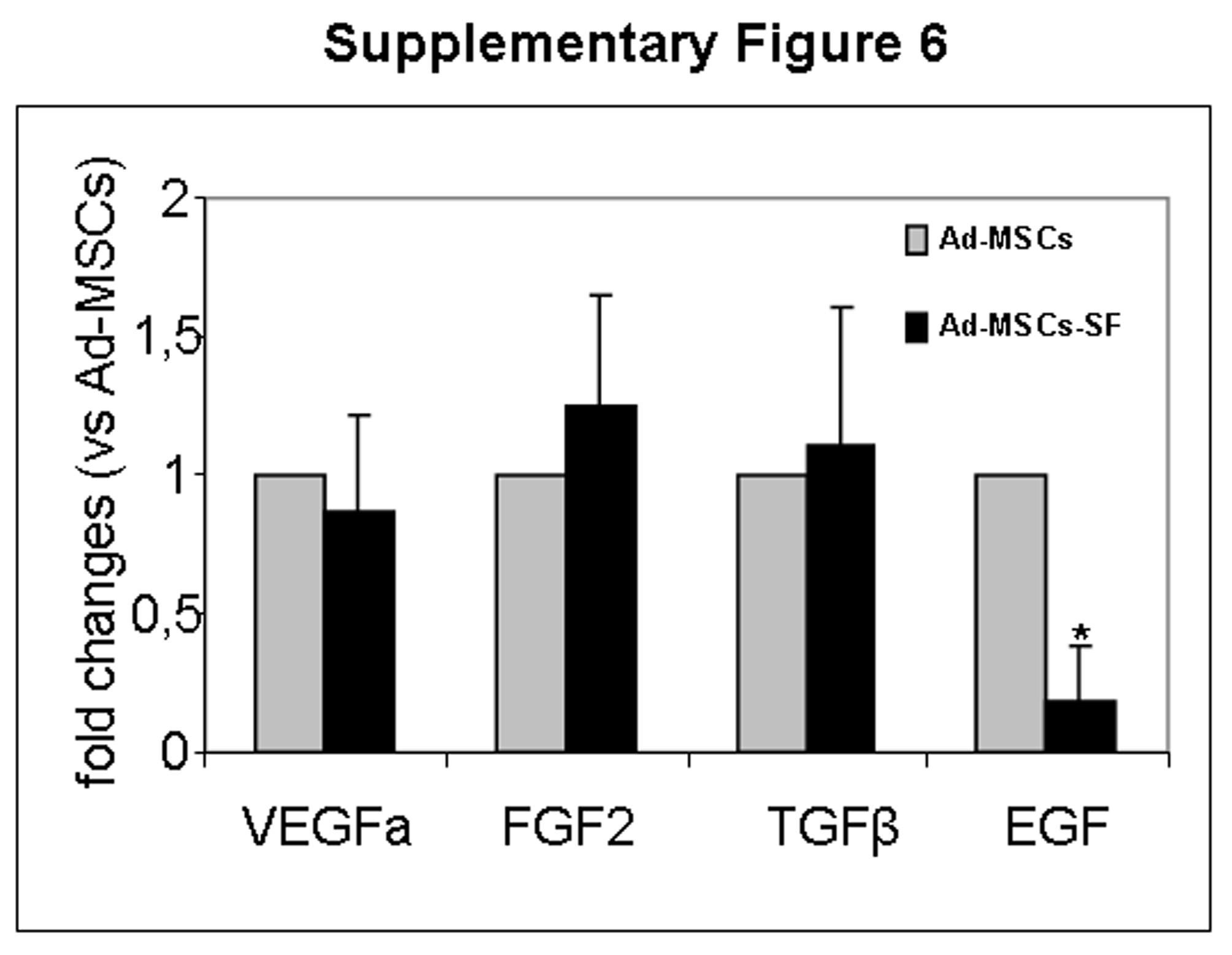

Supplement: Additional file 6: Figure S6 — Angiogenic genes expression of Ad-MSCs seeded on SF patches. RT-PCR was performed to evaluate the gene expression of VEGF, FGF2, TGFβ and EGF on Ad-MSCs cultured on plastic and on SF patches. On the ordinate axis is reported the fold of changes of gene expression of Ad-MSCs on SF patches versus control Ad-MSCs cultured on plastic. The control of gene expression values was considered equal to 1. Relative gene expression was calculated by a comparative method (2-ΔΔCt) using GAPDH as a housekeeping gene. Polymerase chain reactions were carried out in triplicate. Note that in comparing Ad-MSCs grown on plastic versus Ad-MSCs-SF, no significant differences in VEGF, FGF2 and TGFβ were seen; only EGF expression was significantly down-modulated. *P <0.05 versus SF patches. [file scrt396-S6.tiff]
